# Dynamics of dark fermentation microbial communities in the light of lactate and butyrate production

**DOI:** 10.1186/s40168-021-01105-x

**Published:** 2021-07-14

**Authors:** Anna Detman, Daniel Laubitz, Aleksandra Chojnacka, Pawel R. Kiela, Agnieszka Salamon, Albert Barberán, Yongjian Chen, Fei Yang, Mieczysław K. Błaszczyk, Anna Sikora

**Affiliations:** 1 Institute of Biochemistry and Biophysics – Polish Academy of Sciences, Pawińskiego 5a, 02-106 Warsaw, Poland; 2 Department of Pediatrics at Steel Children’s Research Center College of Medicine, University of Arizona, 1501 N. Campbell Avenue, Room 3301, PO Box 245073, Tucson, Arizona 85724-5073 USA; 3 Faculty of Agriculture and Biology, Warsaw University of Life Sciences, Nowoursynowska 159, 02-776 Warsaw, Poland; 4 Institute of Agricultural and Food Biotechnology, Rakowiecka 36, 02-532 Warsaw, Poland; 5 Department of Environmental Science, University of Arizona, 1177 E. 4th Street, P.O. Box 210038, Tucson, Arizona 85721-0038 USA

**Keywords:** Dark fermentation, Lactic acid bacteria, Microbial communities, Nutritional interactions, Lactate, Acetate, Butyrate

## Abstract

**Background:**

This study focuses on the processes occurring during the acidogenic step of anaerobic digestion, especially resulting from nutritional interactions between dark fermentation (DF) bacteria and lactic acid bacteria (LAB). Previously, we have confirmed that DF microbial communities (MCs) that fed on molasses are able to convert lactate and acetate to butyrate. The aims of the study were to recognize the biodiversity of DF-MCs able and unable to convert lactate and acetate to butyrate and to define the conditions for the transformation.

**Results:**

MCs sampled from a DF bioreactor were grown anaerobically in mesophilic conditions on different media containing molasses or sucrose and/or lactate and acetate in five independent static batch experiments. The taxonomic composition (based on 16S_rRNA profiling) of each experimental MC was analysed in reference to its metabolites and pH of the digestive liquids. In the samples where the fermented media contained carbohydrates, the two main tendencies were observed: (i) a low pH (pH ≤ 4), lactate and ethanol as the main fermentation products, MCs dominated with *Lactobacillus*, *Bifidobacterium*, *Leuconostoc* and *Fructobacillus* was characterized by low biodiversity; (ii) pH in the range 5.0–6.0, butyrate dominated among the fermentation products, the MCs composed mainly of *Clostridium* (especially *Clostridium_*sensu_stricto_12), *Lactobacillus*, *Bifidobacterium* and *Prevotella.* The biodiversity increased with the ability to convert acetate and lactate to butyrate. The MC processing exclusively lactate and acetate showed the highest biodiversity and was dominated by *Clostridium* (especially *Clostridium_*sensu_stricto_12). LAB were reduced; other genera such as *Terrisporobacter*, *Lachnoclostridium*, *Paraclostridium* or *Sutterella* were found. Butyrate was the main metabolite and pH was 7. Shotgun metagenomic analysis of the selected butyrate-producing MCs independently on the substrate revealed *C.tyrobutyricum* as the dominant *Clostridium* species. Functional analysis confirmed the presence of genes encoding key enzymes of the fermentation routes.

**Conclusions:**

Batch tests revealed the dynamics of metabolic activity and composition of DF-MCs dependent on fermentation conditions. The balance between LAB and the butyrate producers and the pH values were shown to be the most relevant for the process of lactate and acetate conversion to butyrate. To close the knowledge gaps is to find signalling factors responsible for the metabolic shift of the DF-MCs towards lactate fermentation.

**Video Abstract**

**Supplementary Information:**

The online version contains supplementary material available at 10.1186/s40168-021-01105-x.

## Background

Anaerobic digestion (AD) is a complex and multistep conversion of biomass to methane and carbon dioxide resulting from the metabolic activity and nutritional interactions between many groups of microorganisms. It involves four main stages: hydrolysis of polymeric organic matter to monomers, acidogenesis, acetogenesis and methanogenesis [1–3]. This study focuses on the processes during acidogenesis when the products of hydrolysis are converted to non-gaseous short-chain fatty acids (SCFAs), alcohols, aldehydes and the gases, carbon dioxide and hydrogen [4]. The dominant end-products of the fermentation process determine the type of fermentation. A part of acidogenesis, hydrogen-yielding fermentations (dark fermentation, DF) are considered to be one of the most attractive alternative biological methods of hydrogen (biohydrogen) production. The main types of hydrogen-yielding fermentation under mesophilic conditions, especially from carbohydrate degradation, are acetic/butyric acid fermentation (*Clostridium-*type fermentation) and mixed-acid fermentation (*Enterobacteriaceae*-type fermentation) [3, 5, 6]. Hydrogen can be also produced during the transformation of products other fermentation types. Fermentative biohydrogen production offers the additional advantage of potentially using various waste streams from different industries as feedstock such as the sugar beet industry. Optimization of biohydrogen yield during acidogenesis is challenging and requires a better understanding of the microbial community (MC) dynamics in bioreactors and their metabolic substrate conversion along hydrogen-promoting pathways. In multispecies microbial communities, nutrient utilization is a complex process and frequently involves competition and symbiotic cross-feeding (syntrophy) [7]. The former is when two or more groups of microorganisms compete for a substrate that usually leads to a temporary increase in the relative abundance of one interacting partner over the other. The latter is when the metabolic products yielded by one microbe constitute energy resources or nutrients supporting growth for another one. Therefore, the analysis of nutrient metabolism in fermentative processes should integrate the dynamics of MC composition with metabolic nutrient conversion.

Lactic acid bacteria (LAB) are ubiquitous in the environment; they accompany the plant biomass to anaerobic bioreactors and constitute a relevant component of acidogenic microbial communities (MCs). It is commonly believed that the development of LAB in bioreactors inhibits hydrogen production due to substrate competition and/or excretion of bacteriocins that inhibit the growth of other bacteria. In homolactic fermentation, two molecules of pyruvate formed during glycolysis are converted to lactate; in heterolactic fermentation, one molecule of pyruvate is converted to lactate and the other to ethanol and carbon dioxide [8]. Substrate competition includes the replacement of hydrogen fermentation by lactic acid or ethanol fermentation. A decrease in hydrogen production is observed with a simultaneous increase of lactic acid and ethanol concentrations among non-gaseous fermentation products [9–13].

On the other hand, cross-feeding of lactate involves the conversion of lactate and acetate to butyrate, hydrogen and carbon dioxide. It is a syntrophic nutritional interaction recognized between lactate- and acetate-producing bacteria and butyrate producers. This phenomenon of metabolic interactions between different bacterial groups was described in the gut of many animals including in the human gut. The end product, butyrate, is a crucial molecule necessary in maintaining gut health and homeostasis and serves as an energy source for the colonic epithelial cells [14–17]. Cross-feeding of lactate is also observed in DF bioreactors during the fermentative conversion of organic substrates to biohydrogen in both mesophilic [18–23] and thermophilic conditions [24, 25].

The studies on fermentation of agave bagasse, tequila vinasse and wastewater from nixtamalization supplied data supporting the thesis that cross-feeding of lactate is significant in the MCs of DF bioreactors. The authors postulated that the conversion of lactate and acetate to butyrate is the main pathway of biohydrogen production [19–23]. A specific succession of bacteria was observed in batch experiments. In the first stage, the substrate was processed to acetate and lactate, which were transformed to butyrate and hydrogen in the second stage. The pH was an important factor ensuring balance and syntrophy between lactate and butyrate producers [19–23]. Studies on thermophilic DF of sugarcane vinasse also showed lactate as the primary substrate for biohydrogen production and the relevance of pH in this process [24, 25]. Cross-feeding of lactate was also observed in reduced MCs composed of two components: butyrate-producing *Clostridium beijerinckii* and lactate-producer *Yokenella regensburgei* [26] or butyrate-producing *Clostridium butyricum* and lactate-producer *Sporolactobacillus vineae* [27]. Furthermore, pure cultures of *Clostridium acetobutylicum* [28], *Butyribacterium methylotrophicum* [29], *Clostridium diolis* [30], *Clostridium butyricum* [18] and *Clostridium tyrobutyricum* [31, 32] anaerobically grown in media with acetate and lactate as exclusive carbon sources produced carbon dioxide, hydrogen and butyrate.

Our previous work demonstrated that DF MCs fed molasses under mesophilic conditions are able to convert lactate and acetate to butyrate in batch experiments [18]*.* Here, we propose a logical continuation and extension of the previously published studies aimed at (i) recognition of biodiversity and dynamics of DF MCs able and unable to convert lactate and acetate to butyrate and (ii) definition of the conditions for the process of transformation. We examined batch cultures of DF MCs grown in media containing molasses or sucrose supplemented with lactate and acetate, or a mixture of lactate and acetate without added carbohydrates. The balance between lactic acid bacteria and the butyrate-producing clostridia and the pH values were shown to be the most relevant for the process of lactate and acetate conversion to butyrate. The putative main lactate producers and lactate and acetate utilizers were identified and the presence of the genes encoding enzymes of fermentation pathways in metagenomes was confirmed by KEGG functional analysis.

Since fermentation processes are ubiquitous in anaerobic environments, butyrate and lactate producers are found in anaerobic digesters and among the gut microbiota; the results obtained in this study should interest researchers dealing with studies on both (i) AD and production of gaseous biofuels and (ii) the butyrate production by the gut bacteria.

## Methods

### Experimental set-up for the examination of lactate to butyrate transformation in batch experiments

Tests on the transformation of lactate and acetate to butyrate were conducted in static batch experiments, analogous to those described previously [18], in 250-ml Erlenmeyer flasks for 18 days in a Vinyl Anaerobic Chamber (Coy Laboratory Products, Inc.) without shaking at 30 °C. Five-millilitre samples of MC taken from the DF hydrogen-producing packed bed reactor (PBR1) described previously were used as inoculum [33]. The liquid growth medium (200 ml) was M9 after 10-fold dilution, without glucose, supplemented with 1% sucrose (Chempur Poland) or molasses at the concentration corresponding to 1% sucrose; sodium lactate (VWR Chemicals) 7.41 g/L; sodium acetate (Chempur Poland) 3.5 g/L; and 0.2% yeast extract (BD Biosciences USA). The following combinations of nutrients were tested: molasses (Experiment M); molasses plus sodium lactate (Experiment ML); molasses plus sodium lactate and sodium acetate (Experiment MLA); sodium lactate and sodium acetate (Experiment LA); sucrose plus sodium lactate and sodium acetate (Experiment SLA). All the variants were tested in three independent repetitions designated as A, B and C. Molasses is a by-product of sugar production from sugar beets. It contains 50% sucrose. Other components are water, glucose, fructose, amino acids, mineral salts, betaine, B vitamins, glutamic acid, inositol and nitrogen compounds. In this study, molasses came from the Dobrzelin Sugar Factory, branch of the Polish Sugar Company “Polski Cukier.” Starting pH of all media was 7.0. No additional means of pH control were used. Before inoculation, 4–5 sterile slag pieces were placed in each flask to be covered by bacterial biofilm. Bacterial growth in batch cultures was determined by OD_600nm_ measurements. After every 3 days of incubation, the Erlenmeyer flasks were shaken, the digestive liquids were removed and the respective fresh media for further growth were added. After every passage (on days 3, 6, 9, 12, 15 and 18) the digestive liquids were centrifuged (7000×*g* for 10 min), the supernatants analysed and the pellets used for total microbial DNA isolation as described below. The composition of the selective media and lactate and acetate concentrations was selected based on the data from previous studies [18, 30, 34, 35].

### Analytical methods

The pH of the media and the digestive liquids was measured using a standard pH meter (ELMETRON model CP-502, Poland). Samples were centrifuged (7000×*g* for 10 min, 10 °C) to remove microbial cells and debris, and concentrations of carbohydrates (sucrose, glucose and fructose), short-chain fatty acids, and ethanol were determined. The carbohydrates and ethanol were analysed using high-performance liquid chromatography (HPLC) with refractometric detection (Waters HPLC system: Waters 2695 - Separations Module, Waters 2414 - Refractive Index Detector, a thermostat for column, and 300 × 6.5 mm Sugar Pak I column with guard column). The determination of carbohydrates was carried at 90 °C, and ethanol at 70 °C. The sample (10 μL) was injected onto the column and eluted for 20 min with an isocratic flow of 0.1 mM calcium disodium salt EDTA (0.5 mL/min). Short-chain fatty acids were analysed by HPLC with photometric detection (Waters HPLC system as above, Waters 2996 - Photodiode Array Detector, and 300 × 7.8 mm Aminex HPX-87 H column with guard column at 30 °C). The samples were eluted for 45 min with an isocratic flow (0.6 mL/min) of 4 mM sulphuric acid.

For the statistical analysis of bacterial growth (OD_600nm_), pH of the digestive liquids and the non-gaseous fermentation products, the STATISTICA (version 10.0) computer software (StatSoft, Inc.) was used. All variables were examined for normality and homogeneity of variance. Tukey’s HSD (honestly significant difference) test was applied after ANOVA analysis to compare statistical significance among the variables in experiments. Statistical significance was considered at *p* < 0.05.

### Microbial DNA extraction

The total DNA was isolated from the pellets obtained after centrifugation (see above) of 2-ml samples of the digestive liquids taken after 3, 6, 9, 12, 15 and 18 days of the experiment. From each culture, two samples (duplicates) were taken. DNA was extracted and purified using a DNeasy PowerSoil Pro Kit (Qiagen, Cat No. 47014) according to the manufacturer’s protocol. Cell lysis was done using Vortex-Genie 2 equipped with a Vortex Adapter for 1.5–2-ml tubes (cat. no. 13000-V1-24). DNA was stored at – 20 °C. The final samples of DNA extracted from the two replicates were pooled.

### 16S rRNA amplicon sequencing and data analysis

The hypervariable V4 region of the 16S rRNA gene was amplified from each sample using barcoded reverse primers (806R) and a common forward primer (515F). Both reverse and forward primes were extended with the sequencing primer pads, linkers, and Illumina adapters [36], and with MyFi™ Mix (Bioline Meridian, Cat No. BIO-25050). The PCR was performed on LightCycler 96 (Roche) in the final volume of 40μL. Amplicons were quantified using Quant-It PicoGreen dsDNA Assay kit (ThermoFisher Scientific, Cat No. P7589), according to the manufacturer’s protocol. Equal amount of amplified DNA (240 ng) from each sample were pooled and cleaned using UltraClean PCR Clean-Up Kit (MoBio, Cat No. 12500). Pooled amplicons were diluted and denatured with 0.1N NaOH. The library was sequenced at the Microbiome Core at the Steele Children’s Research Center, University of Arizona, using MiSeq platform (Illumina) and custom primers [36]. Due to the limited sequence diversity among 16S rRNA amplicons, 5% of the PhiX Sequencing Control V3 (Illumina, Cat No. FC-110-3001) made from phiX174, was used to spike the library to increase diversity. The raw sequencing data were demultiplexed using the idemp script (https://github.com/yhwu/idemp). Filtering, dereplication, chimaera identification and merging of paired-end reads were performed with dada2 [37]. The amplicon sequence variant (ASV) taxonomy was assigned using the Ribosomal Database Project (RDP) classifier [38] against SILVA database release 132 [39].

Taxonomic richness and evenness (Shannon and Simpson indices) were calculated and statistical significance within each experiment was calculated using Kruskal-Wallis rank-sum test followed by Dunn’s multiple comparison test with Bonferroni correction (dunn.test R package).

Differences in MCs were evaluated using non-metric multidimensional scaling (NMDS) ordination analysis on Bray-Curtis distances followed by permutational multivariate analysis of variance (PERMANOVA) to analyse the contribution of different metadata variables to MC composition dissimilarities. Also, to investigate and visualize the association between metadata variables and their effect on the species distribution pattern, redundancy analysis was used in *vegan* R package [40]. The obtained results were visualized with a *ggplot2* (ver 3.3.2) package [41] and with heatplus (ver. 3.11) R package [42].

The raw sequences generated in this study have been deposited in NCBI databases with the accession number PRJNA645198.

### Shotgun metagenomic sequencing and data analyses

The libraries for shotgun metagenomic sequencing were constructed for the selected samples from the static batch experiments using QIASeq FX DNA Library Kit (QIAGEN) according to the manufacturer’s protocol. Briefly, 50 ng of DNA from each sample (or pooled samples) was randomly fragmented with FX Enzyme Mix followed by the adapter ligation step. Both i5 and i7 adapters contain unique 8 nucleotide barcodes. After removing free adapters from the reaction with AMPure XP magnetic beads, all individual libraries were amplified by PCR followed by the size selection with 2-step purification (the negative selection followed by the positive selection step) with AMPure XP magnetic beads. The quality and quantity of all libraries were determined with Agilent 4150 TapeStation DNA analyser. The libraries were normalized and pooled, and the sequencing was performed on the Illumina NextSeq 500/550 platform using Illumina 400M HighOutput 300-cycle sequencing chemistry.

Adapter sequences were removed using Cutadapt v. 2.1 [43]. Reads shorter than 50 bp and low-quality bases were removed using Trimmomatic v. 0.38 [44]. The high-quality reads were de novo assembled using Megahit v. 1.1.4 [45]. After discarding assembled contigs shorter than 500 bp, protein-coding genes were predicted using Prodigal v. 2.6 [46]. Paired-end reads were mapped to the genes using BWA v. 0.7.16 [47].

The raw sequences generated in this study have been deposited in NCBI databases with the accession number PRJNA640235.

Gene functional annotations were obtained using the Kyoto Encyclopedia of Genes and Genomes (KEGG) database using GhostKOALA (available at https://www.kegg.jp/ghostkoala/ [48].

## Results

### General characteristics of the MCs in static batch experiments

To examine the capabilities of DF MCs to convert lactate and acetate to butyrate, five independent static batch experiments in three replicates were performed. Each one was inoculated with the same community derived from hydrogen-producing packed bed reactors described previously [18, 49]. The experiments provided different carbon sources as shown in Table 1: molasses (Experiment M), molasses supplemented with lactate (Experiment ML), molasses supplemented with lactate and acetate (Experiment MLA), sucrose supplemented with lactate and acetate (Experiment SLA) and lactate and acetate (Experiment LA). The batch experiments were maintained for 18 days and passaged every 3 days.

**Table 1:** Bacterial growth measured by OD_600nm_ of the digestive liquids after every passage. The data show a mean from three replicates with ± SD. Tukey’s HSD test was applied after ANOVA variance analysis to compare statistical significance; for detailed comparisons, see Additional file 1

Experiment (days)	M (molasses)	ML (molasses + lactate)	MLA (molasses + lactate + acetate)	LA (lactate + acetate)	SLA (sucrose + lactate + acetate)
3	2.8 ± 0.7	3.1 ± 0.3	3.4 ± 0.1	0.8 ± 0.05 ^**b**^	3.2 ± 0.1
6	3.2 ± 0.4	2.4 ± 0.5 ^**a**^	3.6 ± 0.1 ^a^	0.9 ± 0.1 ^**b**^	3.2 ± 0.03
9	3.3 ± 0.4 ^c^	2.3 ± 0.2 ^**a,** c, d^	3.4 ± 0.3 ^a^	1.4 ± 0.2 ^**b**^	3.3 ± 0.1 ^d^
12	2.1 ± 0.1	2.3 ± 0.5 ^**a**^	3.4 ± 0.05 ^a^	1.2 ± 0.3 ^**b**^	2.7 ± 0.4
15	2.8 ± 0.7	3.3 ± 0.01	3.4 ± 0.1	1.1 ± 0.1 ^**b**^	3.3 ± 0.1
18	3.3 ± 0.2	3.2 ± 0.1	3.2 ± 0.2	1.3 ± 0.2 ^**b**^	3.1 ± 0.1

^a^*p* < 0.05 (Experiment ML vs Experiment MLA; Tukey’s HSD test)

^b^0.001 < *p* < 0.005 (Experiment LA vs any other group; Tukey’s HSD test)

^c^*p* < 0.05 (Experiment M vs Experiment ML; Tukey’s HSD test)

^d^*p* < 0.05 (Experiment ML vs Experiment SLA; Tukey’s HSD test)

Bacterial growths measured by OD_600nm_ of the digestive liquids after every passage are presented in Table 1. The results clearly show that sucrose stimulates bacterial growth. The densities were higher (OD_600nm_ after every 3 days ≈ 2–3) when bacteria grew on the media containing sucrose (either from molasses or used a pure additive; Experiments M, ML, MLA and SLA) compared to Experiment LA when lactate and acetate were provided as an exclusive carbon source (OD_600nm_ after every 3 days ≈ 1), *0.001 < p* < 0.005 between LA group and any other group (Tukey’s HSD test; Table 1, Additional file 1). Interestingly, in comparison to molasses and lactate alone (Experiment ML), the addition of acetate in Experiment MLA increased bacterial growth on days 6, 9 and 12 (*p* < 0.05, Tukey’s HSD test). Differences in bacterial growth were also found on day 9 between Experiments M and ML as well as between Experiments M and SLA (*p* < 0.05, Tukey’s HSD test).

**Fig. 1:**
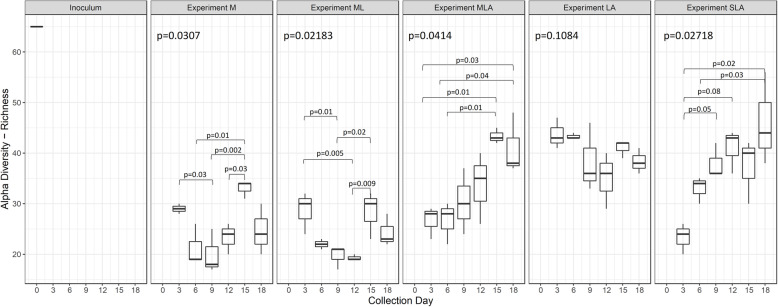
Alpha Diversity (Richness) of the MCs selected in time in the static batch experiments for each collection day, except day 0, which is an inoculation day. The lower and upper hinges represent the first and third quartiles respectively. The whiskers extend to the largest and lowest values. The middle line represents the median value. Dots represent individual samples. For statistical analysis, the Kruskal-Wallis rank-sum test followed by Dunn’s multiple comparison test was used

**Fig. 2:**
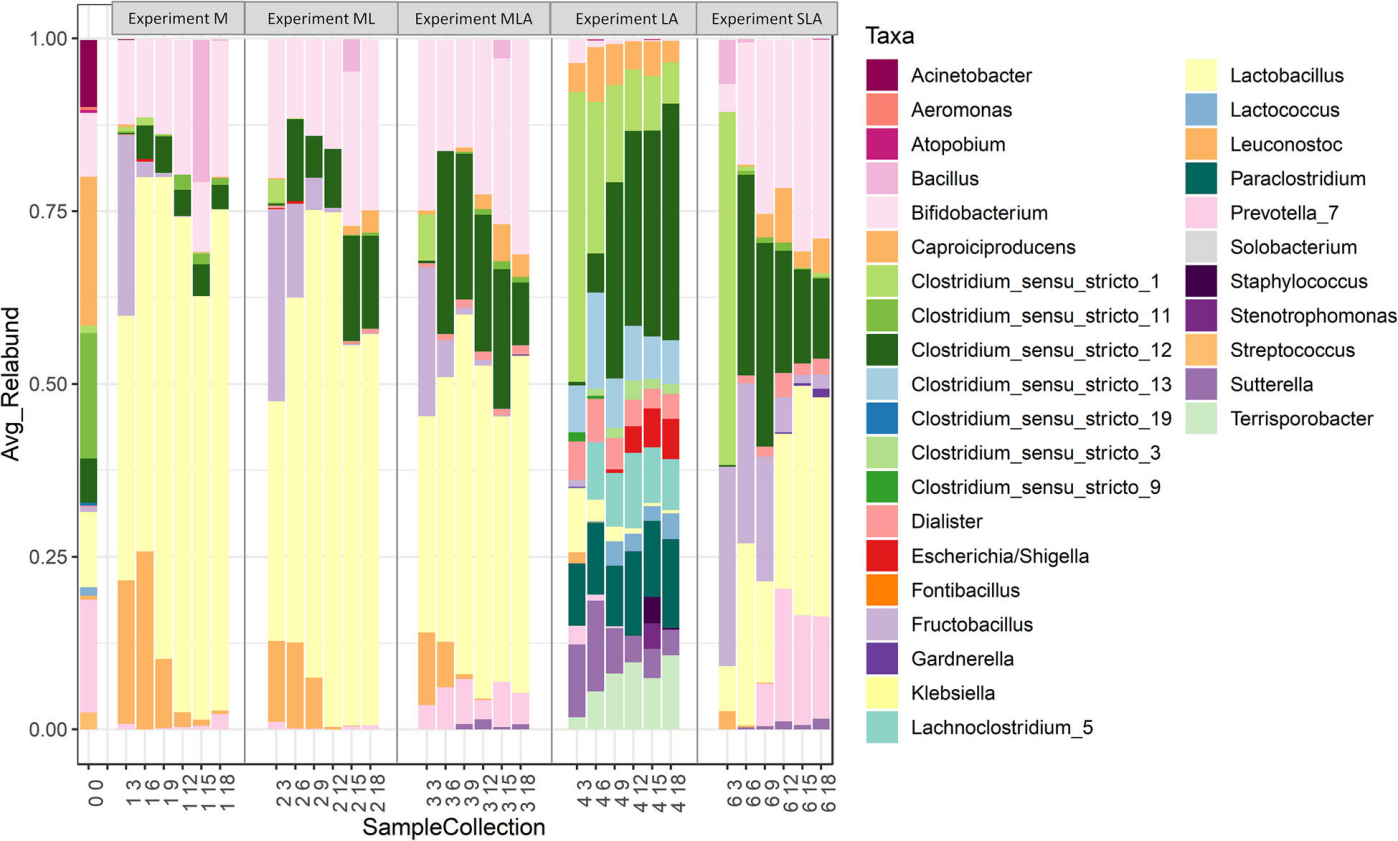
Taxonomic composition (genus level) of the MCs selected in the batch experiment based on hypervariable V4 region of the 16S rRNA gene, sequenced on MiSeq platform (Illumina). The taxonomy was assigned using the RDP classifier against the SILVA database. All taxa with relative abundance lower than 0.1% were removed

**Fig. 3:**
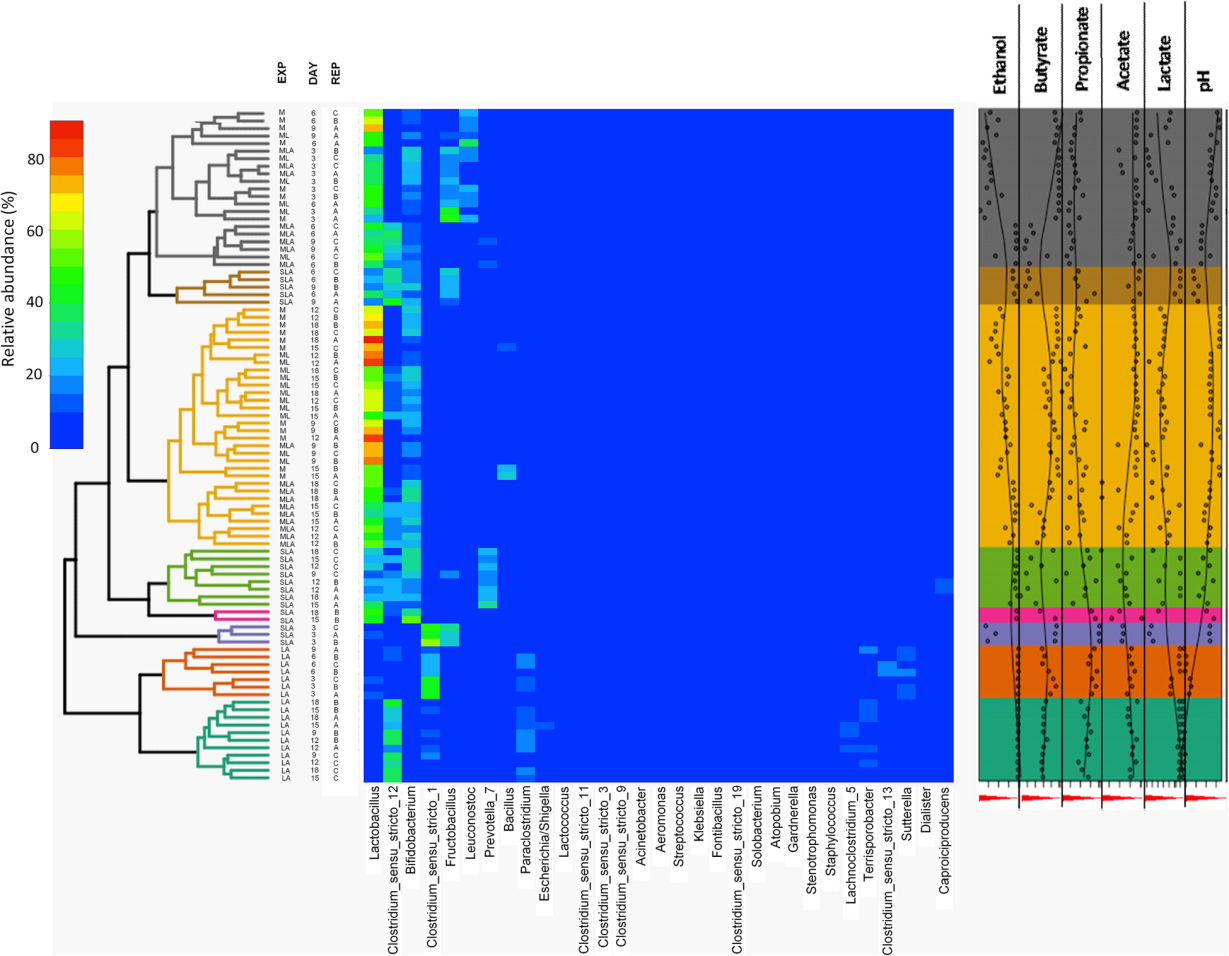
Heatmap showing the relative abundance of genera in the individual Experiments for all timepoints and annotation with measured metabolites and pH. The heatmap was generated in R (Heatplus package, annHeatmap2 function) using the relative abundance of the observed genera. For clarity, the “Inoculum” sample and all genera with summarized relative abundance lower than 0.1% were removed. Rows were clustered using average linkage hierarchical clustering based on the Bray–Curtis dissimilarity matrix of the dataset (“vegdist” from the vegan package)

**Fig. 4:**
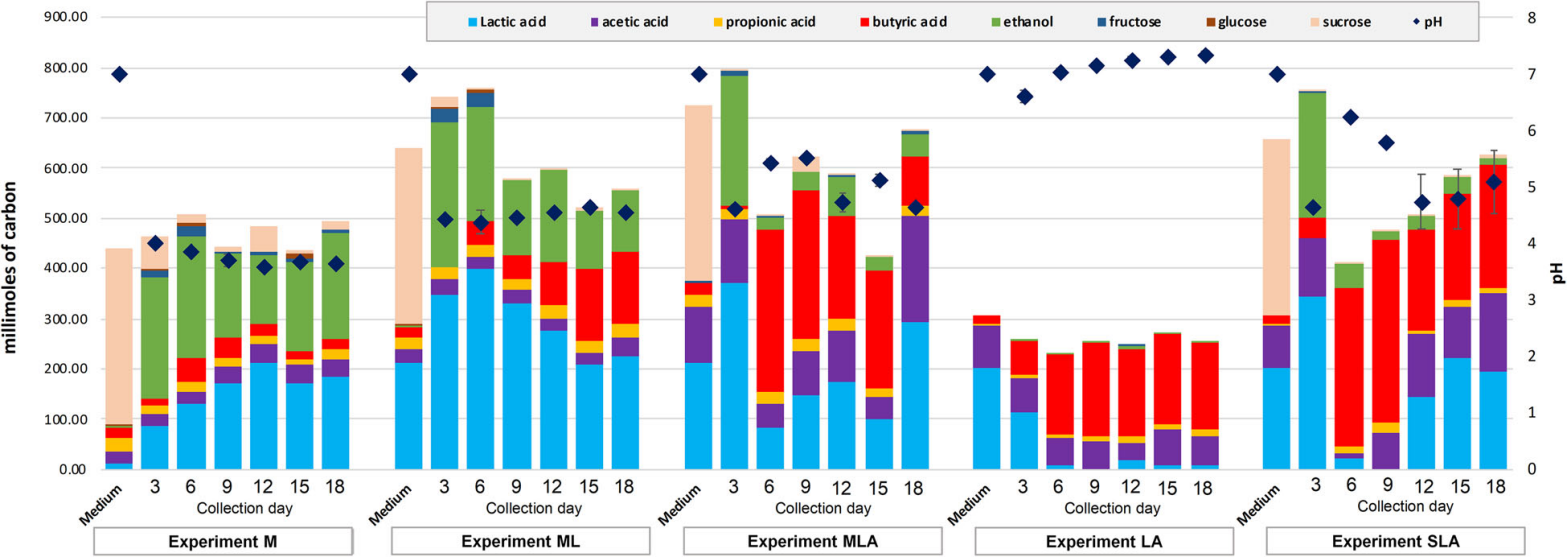
Non-gaseous fermentation products expressed in millimoles of carbon (bars, left axis) and pH (diamonds, right axis) of the digestive liquids from batch experiments presented in time for each experiment. The values are a mean from 3 replicates; for each, the analyses were performed in duplicate. The composition of fermentation products was analysed using high-performance liquid chromatography. The pH values are a mean from 3 replicates ± SD. For detailed data, see Additional file 4

Biodiversity and microbial changes in all the experiments were analysed by sequencing of the 16S V4 amplicon profiling. A total of 119 samples were sequenced in one MiSeq run, and 7431 ASVs were detected. After chimaera identification and removal, 93.15% ASVs remained. 29 samples from an unrelated project were filtered out, and the remaining 90 samples were further analysed. For detailed taxonomic assignments see Additional file 2. All negative controls for the V4 amplification by PCR (collection day 0 for each experiment) did not show any amplification and these controls were removed from analysis during the quality control steps due to insufficient number of reads. Alpha diversity analysis revealed that the MCs are moderately rich in taxa, and that communities grown in media supplemented with molasses only or molasses and lactate (Experiments M and ML) had the lowest diversity as compared to the inoculum alone or to other groups (Fig. 1, Additional file 3). Taxonomic composition of each experimental MC (Figs. 2 and 3) was analysed in reference to its metabolites, i.e. non-gaseous fermentation products and pH of the digestive liquids (Figs. 3 and 4, Table 2).

**Table 2:** Characteristics of the digestive liquids. The data show a mean from three replicates with ± SD, for each the analyses were performed in duplicate. Tukey’s HSD test was applied after ANOVA variance analysis to compare statistical significance; for detailed comparisons see Additional file 4

Experiment	Collection day	pH	Acetic acid	Lactic acid	Propionic acid	Butyric acid	Ethanol
			g/L				
Experiment M	3	4.0 ± 0.0	0.8 ± 0,2	2.6 ± 0.4 ^**a**^	0.4 ± 0.1	0.3 ± 0.5	5.5 ± 1.9
	6	3.9 ± 0.1 ^**#**^	0.8 ± 0,1	3.9 ± 0.4 ^**b**^	0.5 ± 0.1	1.0 ± 0.1 ^**$**^	5.6 ± 1.5 ^**@**^
	9	3.7 ± 0.1 ^##^	1.0 ± 0.2	5.2 ± 0.3 ^**a**^	0.4 ± 0.1	0.9 ± 0.6 ^**$$**^	3.8 ± 2.1 ^**@@**^
	12	3.6 ± 0,03	1.2 ± 0.3	6.3 ± 0.7 ^**a, b**^	0.5 ± 0.1	0.5 ± 0.3	3.1 ± 0.6
	15	3.7 ± 0.03	1.0 ± 0.2	5.2 ± 0.7 ^**a**^	0.3 ± 0.03	0.4 ± 0.2	4.1 ± 0.4
	18	3.6 ± 0.03	1.0 ± 0.1	5.6 ± 0.2 ^**a, b**^	0.5 ± 0.1	0.4 ± 0.2	4.9 ± 0.5
Experiment ML	3	4.4 ± 0.01	1.0 ± 0.2	10.4 ± 1.5	0.5 ± 0.1	0.03 ± 0.01 ^**d**^	6.7 ± 1.1 ^**e**^
	6	4.4 ± 0.2 ^**#**^	0.7 ± 0.4	12.0 ± 2.1 ^**c, &**^	0.6 ± 0.1	1.1 ± 1.7 ^**$**^	5.2 ± 0.7 ^**@**^
	9	4.5 ± 0.1 ^##^	0.8 ± 0.1	10.0 ± 0.5 ^**&&**^	0.5 ± 0.1	1.1 ± 1.0 ^**$$**^	3.4 ± 1.1 ^**e, @@**^
	12	4.5 ± 0.03	0.7 ± 0.2	8.3 ± 2.5	0.7 ± 0.1	1.9 ± 0.6	4.2 ± 1.5
	15	4.6 ± 0.04	0.7 ± 0.2	6.3 ± 0.6 ^**c**^	0.6 ± 0.1	3.2 ± 0.8 ^**d**^	2.7 ± 0.6 ^**e**^
	18	4.6 ± 0.04	1.1 ± 0.3	6.8 ± 1.2 ^**c**^	0.7 ± 0.1	3.2 ± 0.7 ^**d**^	2.8 ± 1.1 ^**e**^
Experiment MLA	3	4.6 ± 0.01 ^**f**^	3.8 ± 0.4	11.2 ± 0.9	0.5 ± 0.1	0.2 ± 0.03 ^**g**^	5.9 ± 0.6 ^**h**^
	6	5.4 ± 0.1 ^**f , #**^	1.4 ± 0.1	2.5 ± 0.8 ^**&**^	0.6 ± 0.01	7.1 ± 0.5 ^**g, $**^	0.6 ± 0.04 ^**h, @**^
	9	5.5 ± 0.01 ^**f,** ##^	2.7 ± 1.4	4.4 ± 6.5 ^**&&**^	0.6 ± 0.02	6.5 ± 3.5 ^**g, $$**^	0.9 ± 0.7 ^**h, @@**^
	12	4.7 ± 0.2	3.1 ± 0.9	5.2 ± 1.8	0.6 ± 0.1	4.5 ± 0.9	1.8 ± 0.8 ^**h**^
	15	5.1 ± 0.1	1.4 ± 0.6	2.9 ± 2.3	0.4 ± 0.1	5.2 ± 1.6	0.6 ± 0.1 ^**h**^
	18	4.6 ± 0.03	6.3 ± 1.8	8.8 ± 2.2	0.5 ± 0.1	2.2 ± 0.4	1.0 ± 0.1 ^**h**^
Experiment LA	3	6.6 ± 0.1	2.1 ± 0.5	3.4 ± 0.2 ^**i**^	0.1 ± 0.04	1.5 ± 0.7 ^**j**^	0.1 ± 0.03
	6	7.0 ± 0.1 ^**#**^	1.7 ± 0.5	0.2 ± 0.2 ^**I, &**^	0.2 ± 0.1	3.5 ± 0.8 ^**j, $**^	0.1 ± 0.1 ^**@**^
	9	7.2 ± 0.1 ^##^	1.7 ± 0.1	0.02 ± 0.01 ^**I, &&**^	0.2 ± 0.1	4.2 ± 0.2 ^**j, $$**^	0.04 ± 0.04 ^**@@**^
	12	7.2 ± 0.05	1.1 ± 0.4	0.5 ± 0.3 ^**i**^	0.3 ± 0.1	3.8 ± 0.7 ^**j**^	0.2 ± 0.03
	15	7.3 ± 0.03	2.1 ± 0.2	0.2 ± 0.2 ^**i**^	0.2 ± 0.0	4.0 ± 0.1 ^**j**^	0.1 ± 0.04
	18	7.3 ± 0.1	1.7 ± 1.0	0.2 ± 0.15 ^**i**^	0.4 ± 0.04	3.8 ± 0.5 ^**j**^	0.1 ± 0.03
Experiment SLA	3	4.6 ± 0.03 ^**k**^	3.4 ± 0.1	10.4 ± 0.7	0.02 ±0.01	0.9 ± 0.2 ^**l**^	5.8 ± 1.0 ^**m**^
	6	6.2 ± 0.04 ^**k, #**^	0.3 ± 0.1	0. 7 ± 1.1 ^**&**^	0.4 ± 0.2	7.0 ± 1.3 ^**l, $**^	1.1 ± 0.2 ^**m, @**^
	9	5.8 ±0.03 ^**k,** ##^	2.2 ± 0.7	0.02 ± 0.01 ^**&&**^	0.5 ± 0.2	8.0 ± 0.6 ^**l, $$**^	0.4 ± 0.1 ^**m, @@**^
	12	4.7 ± 0.5	3.7 ± 0.7	4.4 ± 3.9	0.2 ± 0.1	4.4 ± 3.6	0.7 ± 0.1 ^**m**^
	15	4.8 ± 0.5	3.1 ± 2.1	6.7 ± 6.9	0.3 ± 0.2	4.7 ± 3.2	0.8 ± 0.8 ^**m**^
	18	5.1 ± 0.6	4.7 ± 2.5	5.9 ± 5.1	0.3 ± 0.1	5.4 ± 4.1	0.3 ± 0.1 ^**m**^

Results of the Tukey’s HSD test:

^**a**^ day 3 vs day 9, *p* = 0.0005; day 3 vs day 12, *p* = 0.0002; day 3 vs day 15, *p* = 0,0005; day 3 vs day 18, *p* = 0,0003

^**b**^ day 6 vs day 12, *p* = 0.0009; day 6 vs day 18, *p* = 0.02

^**c**^ day 6 vs day 15, *p* = 0,01; day 6 vs day 18, *p* = 0,02

^**d**^ day 3 vs day 15, *p* = 0.01; day3 vs day 18, *p* = 0.01

^**e**^ day 3 vs day 9, *p* = 0.02; day 3 vs day 15 *p* = 0.006; day 3 vs day 18, *p* = 0.007

^**f**^ day 3 vs day 6, *p* = 0.0002; day vs day 9, *p* = 0.0002

^g^ day 3 vs day 6, *p* = 0.002; day vs day 9, *p* = 0.004

^**h**^ day 3 vs days 6, 9, 12, 15 and 18, *p* = 0.0002

^**i**^ day 3 vs day 6, *p* = 0.0002; day 3 vs day 9, *p* = 0.0002; day 3 vs day 12, *p* = 0.0002; day 3 vs day 15, *p* = 00002; day 3 vs day 18, *p* = 0.0002

^**j**^ day 3 vs day 6, *p* = 0.009; day 3 vs day 9, *p* = 0.001; day 3 vs day 12, *p* = 0.003; day 3 vs day 15, *p* = 0.002; day 3 vs day 18, *p* = 0.003

^**k**^ day 3 vs day 6, *p* = 0.002; day vs day 9, *p* = 0.02

^**l**^ day 3 vs day 6, *p* = 0.0004; day vs day 9, *p* = 0.0003

^**m**^ day 3 vs days 6, 9, 12, 15 and 18, *p* = 0.0002

^**#**^ pH after 6 days: Exp. 1/M vs Exp. 2/ML, *p* = 0.001; Exp. 1/M vs Exp 3/MLA, 4/LA, 6/SLA, *p* = 0.0002; Exp. 2/ML vs Exp. 3/MLA, Exp. 4/LA, Exp. 6/SLA, *p* = 0.0002; Exp. 3/MLA vs Exp. 4/LA, Exp. 6/SLA, *p* = 0.0002; Exp. 4/LA vs Exp. 6/SLA, *p* = 0.0002

^**##**^ pH after 9 days: Exp. 1/M vs Exp. 2/ML, Exp 3/MLA, 4/LA, 6/SLA, *p* = 0.0002; Exp. 2/ML vs Exp. 3/MLA, Exp. 4/LA, Exp. 6/SLA, *p* = 0.0002; Exp. 3/MLA vs Exp. 4/LA, *p* = 0.0002; Exp. 3/MLA vs Exp. 6/SLA, *p* = 0.0008; Exp. 4/LA vs Exp. 6/SLA, *p* = 0.0002

^**$**^ butyrate concentration after 6 days: Exp. 1/M vs Exp. 3/MLA, Exp. 6/SLA, *p* = 0.0004; Exp. 2/ML vs Exp. 3/MLA, Exp. 6/SLA, *p* = 0.0004; Exp. 3/MLA vs Exp. 4/LA, *p* = 0.01; Exp. 4/LA vs Exp. 6/SLA, *p* = 0.01

^**$$**^ butyrate concentration after 9 days: Exp. 1/M vs Exp. 3/MLA, *p* = 0.01; Exp. 1/ML vs Exp. 6/SLA, *p* = 0.003; Exp. 2/ML vs Exp. 3/MLA, *p* = 0.02; Exp. 2/ML vs Exp. 6/SLA, *p* = 0.003

^**&**^ lactate concentration after 6 days: Exp. 2/ML vs Exp. 3/SLA, Exp. 4/LA, Exp. 6/SLA, *p* = 0.0003

^**&&**^ lactate concentration after 9 days: Exp. 2/ML vs Exp. 4/LA, Exp. 6/SLA, *p* = 0.03

^**@**^ ethanol concentration after 6 days: Exp. 1/M vs Exp. 3/MLA, Exp. 4/LA, *p* = 0.0002; Exp. 1/M vs Exp. 6/SLA, *p* = 0.0003; Exp. 2/ML vs Exp. 3/MLA, *p* = 0.0003; Exp. 2/ML vs Exp. 4/LA, *p* = 0.0002; Exp. 2/ML vs Exp. 6/SLA, *p* = 0.0005

^**@@**^ ethanol concentration after 9 days: Exp. 1/M vs Exp. 4/LA, Exp. 6/SLA, *p* = 0.02; Exp. 2/ML vs Exp. 4/LA, *p* = 0.03; Exp. 2/ML vs Exp. 6/SLA, *p* = 0.05

### Analysis of metabolites and MC composition after the initial 3 days of fermentation

After the initial 3 days of fermentation, we found no statistically significant differences in the concentration of the analysed non-gaseous fermentation products among the batch experiments where growth media contained sucrose (either as a component of molasses or pure sucrose; Experiments M, ML, MLA and SLA). The concentration of butyrate was low (< 1 g/L; Table 2, Fig. 4, Additional file 4). The main fermentation products were ethanol (5.5–6.7 g/L) and lactate. After the initial 3 days of fermentation with molasses only (Experiment M), lactate concentration was lowest at 2.6 g/L. In the case of Experiments ML, MLA and SLA, the concentration of lactate in the digestive liquids (10.4–11.2 g/L) was the sum of that in the media and as a product of sucrose fermentation. Fermentation of sucrose (Experiments M, ML, MLA, and SLA) resulted in similar pH of the digestive liquids (4.0, 4.4, 4.6 and 4.6, respectively). Detailed data for each metabolite and time point are presented in Table 2 and Additional file 4.

After the initial 3 days of fermentation, communities grown on the media containing molasses (Experiments M, ML, and MLA) were composed primarily of *Lactobacillus* (38.3% ± 15.6, 34.7% ± 4.4, 38.3% ± 5.1, respectively), *Fructobacillus* (26.1% ± 17.6, 27.8% ± 12.0, 21.5% ± 5.8, respectively), *Bifidobacterium* (12.2% ± 3.0, 20.1% ± 7.8, 24.7% ± 1.4, respectively), and *Leuconostoc* (20.7% ± 3.3, 11.7% ± 3.2, 10.6% ± 3.9, respectively), with a smaller proportion of *Clostridium* sensu stricto 1 (0.6% ± 0.5, 3.3% ± 2.2, 6.7% ± 1.2, respectively). Compared to Experiments M, ML and MLA, MC grown with pure sucrose (Experiment SLA) showed lower contribution of *Bifidobacterium* (4.0% ± 3.4; *p* = 0.036, *p* = 0.05, and *p* = 0.003, respectively), *Lactobacillus* (6.5% ± 5.8; *p* = 0.057, *p* = 0.003, *p* = 0.03, respectively) and *Leuconostoc* (2.6% ± 1.3; *p* = 0.005, *p* = 0.025, *p* = 0.062, respectively), and the community became dominated by *Clostridium* sensu stricto 1 (51.1% ± 7.5; *p* = 0.007, *p* = 0.005, *p* = 0.008, respectively). However, at this stage of fermentation, this genus did not appear to correlate with butyrate production. The relative abundance of *Fructobacillus* (28.9% ± 3.2) in SLA community was comparable to the communities in M, ML, and MLA (*p* = 0.81, *p* = 0.89, *p* = 0.14, respectively) (Figs. 2 and 3, Additional file 2).

The dynamics of the fermentation process were followed over four additional passages until 18 days post-inoculation and showed considerable differences between experimental groups. These are discussed in detail in the following sections.

### Dynamics of fermentation processes with molasses only

When molasses were fermented without exogenous SCFAs, the pH of the digestive liquids after 6 days dropped below 4 and remained in the 3.6–3.9 range (Table 2, Fig. 4, Additional file 4). During the whole experiment, the main non-gaseous fermentation products were ethanol and lactate. Lactate was the only metabolite that significantly changed over time (ANOVA, *p* = 0.000015), with a gradual increase from day 3 to day 18 (2.6, 3.9, 5.2, 6.3, 5.2 and 5.6 g/L, respectively). The results of the detailed statistical comparisons are presented in Table 2 and Additional file 1. Between 6 and 18 days, the concentrations of ethanol remained relatively stable at 5.6, 3.8, 3.1, 4.1 and 4.9 g/L. The concentrations of butyrate and acetate were low (≤ 1 g/L) throughout the experiment (Table 2, Fig. 4).

The overall biodiversity was low compared to the inoculum and showed changes over time, although without a clear trend (Fig. 1). MC was dominated by *Lactobacillus* (54.2% ± 7.8, 69.8% ± 5.3, 71.7 ± 11.6, 61.3 ± 11.9 and 72.6 ± 12.0, respectively after 6, 9, 12, 15 and 18 days), with *Bifidobacterium* as the second most abundant genus (11.3% ± 3.7, 15.7% ± 6.0, 19.5% ± 9.6, 10.1% ± 2.2 and 19.7% ± 7.9, respectively after 6, 9, 12, 15 and 18 days) (Fig. 2). The *Leuconostoc* genus was a significant component of the MC on day 3 and 6 (20.7% ± 3.3 and 25.8% ± 9.7, respectively), but its relative abundance started to decline on day 9 and onwards to eventually constitute a minor genus (10.1% ± 8.1 on day 6, 2.1% ± 1.5 on day 6, and < 1% on days 15 and 18). The relative abundance of the genera *Clostridia* sensu stricto was generally low (5–6%); among them, *Clostridium* sensu stricto 12 dominated (4.8% ± 3.8%, 5.3% ± 2.8%, 3.8% ± 1.3%, 4.6% ± 0.5% and 3.5% ± 1.3% after 6, 9, 12, 15 and 18 days, respectively)***.***

### Dynamics of fermentation processes with molasses supplemented with lactate

After the addition of lactate, the pH of the digestive liquids after the first passage (days 6–18) remained in the range 4.4–4.6. The concentrations of ethanol decreased steadily (ANOVA, *p* = 0.004) from 6.7 g/L on day 3 to 3.4, 2.7 and 2.8 g/L on days 9, 15 and 18, respectively. Concentration of lactate varied over time (ANOVA, *p* = 0.006)*.* It peaked on day 6 at 12 g/L and decreased to 6.3 and 6.8 g/L on days 15 and 18, respectively. The concentration of butyrate gradually increased (ANOVA, *p* = 0.006) from 0.03 g/L on day 3 to 3.2 g/L on days 15 and 18. The concentration of acetate remained low and steady (≤ 1 g/L) throughout the experiment (Table 2, Fig. 2, Additional files 1 and 4).

Supplementation of molasses with lactate as a source of carbon overall did not change the richness of the bacterial community, which remained similar to that in Experiment M, with molasses as a sole source of carbon. After inoculation, we observed a transient drop of richness until day 12 followed by the restored number ASVs to the original level on day 15 followed by a not statistically significant decline on day 18 (Fig. 1). Similar to the Experiment M, the MCs were dominated with *Lactobacillus* (49.9% ± 5.1, 67.7% ± 15.9, 74.5% ± 9.7, 55.1% ± 8.4 and 56.7% ± 7.1, after 6, 9, 12, 15 and 18 days, respectively) and *Bifidobacterium* (11.4% ± 0.8, 13.9% ± 5.6, 15.7% ± 6.0, 22.8% ± 2.3 and 24.7% ± 6.2, after 6, 9, 12, 15 and 18 days, respectively). The relative contribution of the *Leuconostoc* genus decreased over time (12.5% ± 6.3%, 7.4% ± 7.0% and < 1%, on day 6, 9 days and on and beyond day 12; *p* = 0.016, Kruskall-Walllis test). The *Fructobacillus* genus followed a similar pattern (13.6% ± 12.8%, 4.7% ± 5.7% and < 1%, on day 6, 9 days and on and beyond day 12; *p* = 0.015, Kruskall-Walllis test). The relative abundance of *Clostridium* sensu stricto 12 genus showed a slight decline from day 3 to 6, followed by recovery and modest expansion (11.9% ± 13.8%, 6.1% ± 2.7%, 8.5% ± 4.1%, 15.2% ± 5.4% and 13.4% ± 3.1%, on day 6, 9, 12, 15 and 18, respectively; *p = 0.06*, Kruskall-Walllis test). Corresponding with increased butyrate synthesis on days 15 and 18, the MC showed relative expansion of *Lactobacillus*, *Clostridium* sensu stricto 12 and *Bidifidobacterium* genera (Fig. 2). Among minor genera, the contribution of *Caproiciproducens* increased to 1.3% ± 0.9% and 3.2% ± 1.2%, after 15 and 18 days, respectively (*p* = 0.011, Kruskall-Walllis test).

### Dynamics of fermentation processes with molasses or sucrose supplemented with lactate

In the MLA and SLA Experiments, the media contained molasses as a source or sucrose or pure sucrose, both supplemented with lactate and acetate. These two experiments are described together due to similar tendencies observed, which reflects the dominant effect of lactate/acetate supplementation over the source of sucrose (Figs. 3 and 4, Table 2, Additional files 1 and 4). In the MLA Experiment, the pH of the digestive liquids changed from 4.6 on day 3 to 5.4 and 5.5 on days 6 and 9 (*p* = 0.0002, *p* = 0.0002, respectively; Tukey’s HSD test). In the SLA Experiment, the pH changed from 4.6 on day 3 to 6.2 and 5.8 on days 6 and 9 (*p* = 0.002, *p* = 0.02, respectively; Tukey’s HSD test). In the MLA Experiment, the pH increase was associated with increased butyrate concentration, from 0.2 g/L on day 3 to 7.1 and 6.5 g/L on days 6 and 9 (*p* = 0.002, *p* = 0.004, respectively; Tukey’s HSD test). In the SLA Experiment, the concentration of butyrate increased from 0.9 g/L on day 3 days to 7.0 g/L and 8.0 g/L on days 6 and 9 (*p* = 0.0004, *p* = 0.0003, respectively; Tukey’s HSD test). During longer fermentation (days 12, 15 and 18), butyrate remained an abundant fermentation product and pH was maintained at ca. 5. On day 6 and onwards, ethanol concentration decreased and it became a minor metabolite compared to samples collected on day 3 in either MLA or SLA Experiment (*p* = 0.0002, Tukey’s HSD test).

The biodiversity measured by richness index in both MLA and SLA Experiments increased over time (Fig. 1). The MCs (Figs. 2 and 3) associated with the highest butyrate production in Experiments MLA and SLA, were dominated by *Bifidobacterium* (16–30% for both Experiments), *Clostridium* sensu stricto 12 (20–30% for both Experiments), *Lactobacillus* (40-50% for Experiment MLA and 20–30% for Experiment SLA), *Prevotella* (up to 6% in Experiment MLA and above 15% in Experiment SLA on day 12 and 18), and *Caproiciproducens*. In the MLA and SLA Experiments, we detected a higher contribution of *Prevotella* in comparison to the other culture conditions. In the MLA Experiment, the contribution of the *Leuconostoc* and *Fructobacillus* genera decreased over time, from 6.6% ± 2.2 and 5.3% ± 0.4 after 6 days, respectively, to below 1% from day 9 onwards. In the SLA Experiment, *Leuconostoc* was a minor genus whereas *Fructobacillus* also decreased in time (23.2% ± 2.5, 18.0% ± 4.7, 5.1% ± 1.8, 1.2% ± 0.3 and 2.1% ± 2.9) on days 6, 9, 12, 15 and 18, respectively (*p* = 0.007 and *p* = 0.009 for *Leuconostoc* and *Fructobacillus*, respectively; Kruskall-Wallis test). In both experiments, we observed an increasing contribution of *Caproiciproducens* genus (MLA: < 1%, 2.1% ± 1.8%, 5.4% ± 0.6%, 4.6% ± 1.8%, on days 9, 12, 15, 18, respectively; SLA: < 1%, 3.4% ± 2.4%, 7.9% ± 5.2%, 2.3% ± 2.0%, 5.0% ± 1.6%, on days 6, 9, 12, 15 and 18, respectively; *p* = 0.02 for either genus, Kruskall-Wallis test).

Clustering analysis of each experiment revealed that some individual experimental replicates differed from the other counterparts and were more similar to those from other experiments. For example, replicate B after the 3rd passage (9 days) from Experiment MLA grouped with the samples collected after the 3rd passage (9 days) from Experiment ML. Other examples are replicates from Experiment SLA after the 4th, 5th and 6th passages, respectively, 12, 15 and 18 days (Fig. 3). We had no logical explanation and thus no reason to exclude these replicates from the analysis.

### Dynamics of fermentation of lactate and acetate as the main carbon sources

A distinct scenario occurred for Experiment LA when the source of carbon was limited to lactate and acetate (Table 2 and Additional files 1 and 4). The pH of the digestive liquids was maintained at approximately 7, and the lowest reached value of 6.6 was observed after the first 3 days. Since the second passage (after 6 days), lactate was efficiently utilized (90–100%) and the dominant fermentation product, butyrate, was maintained at a similar level during the whole experiment at around 4 g/L (Table 2). Acetate was detected as the second component (1–2 g/L) of the digestive liquids, whereas propionate (0.2–0.4 g/L) and ethanol (0.05–0.2 g/L) were detected as minor products.

The initial richness of the LA community was higher compared to other experiment conditions and did not significantly changed over time (Fig. 1). Taxonomic analysis to some extent reflected this result (Fig. 2). The genera *Clostridium* sensu stricto constituted on average 45–50%. *Clostridium* sensu stricto 12 increased over time (0.5% ± 0.3%, 5.7% ± 4.9%, 28.4% ± 12.6%, 28.2% ± 2.3%, 29.8% ± 8.1% and 34.2% ± 1.4% after 3, 6, 9, 12, 15 and 18 days, respectively, Kruskall-Wallis test *p* = 0.034) whereas *Clostridium* sensu stricto 1 decreased (41.9% ± 0.6%, 21.9% ± 0.8%, 14.0% ± 3.0%, 8.9% ± 2.3%, 7.9% ± 3.1% and 6.0% ± 1.4% after 3, 6, 9, 12, 15 and 18 days, respectively, Kruskall-Wallis test *p* = 0.012) in time. *Clostridium* sensu stricto 1 was a dominant genus after the first 3 days when conversion of lacate to butyrate was on the lowest level (Figs. 3 and 4, Table 2). The relative abundance of *Clostridium* sensu stricto 13 was also high, but maintained at a similar level over time (6.8% ± 1.5%, 13.9% ± 3.5%, 7.2% ± 1.5%, 7.9% ± 1.6%, 6.1% ± 2.1% and 6.3% ± 1.1%, respectively after 3, 6, 9, 12, 15 and 18 days). Compared to the other experiments, *Lactobacillus* (9.2% ± 4.7%, 3.1% ± 2.4%, 2.1% ± 2.0% after 3, 6, 9 days, respectively, and < 1% since the 12th day), *Fructobacillus* (1.0% ± 0.2% after 3 days and and < 1% since the 6th day), *Bifidobacterium* (3.5% ± 0.4% after 3 days and and < 1% since the 6th day) and *Leuconostoc* (1.6% ± 0.4% after 3 days and and < 1% since the 6th day) were significantly reduced. Contribution of the following genera increased: *Terrisporobacter* (5–10%), *Sutterella* (5–10%), *Paraclostridium* (up to 10%), *Lachnoclostridium* (up to 10%), *Escherichia* (up to 5%) and *Dialister* (4–6%).

### Summary of the static batch experiments and redundancy analysis

Detailed statistical comparison of the pH and metabolite (ethanol, butyrate, propionate and lactate) formation between Experiments M, ML, MLA, LA and SLA are depicted in Additional file 1. For simplicity, in this section, we focus on the results from days 6 to 9. The pH values were significantly different among all experiments (0.001 > *p* > 0.0002, Tukey’s HSD test; Additional file 1) with the lowest pH recorded in Experiment M (molasses only; Table 2). Butyrate synthesis in Experiments M and ML was significantly lower than in Experiments MLA, SLA and LA (0.02 > *p* > 0.0004, Tukey’s HSD test; Additional file 1, Table 2). A reverse tendency was observed for ethanol production which was higher in Experiments M and ML compared to MLA, SLA and LA (0.05 > *p* > 0.0005, Tukey’s HSD test). Detected lactate concentrations in the digestive liquids from Experiments ML, MLA, SLA and LA, where lactate was added to the media, clearly show more efficient utilization of lactate in Experiments MLA, SLA and LA as compared to Experiment ML (0.04 > *p* > 0.0004, Tukey’s HSD test; Additional files 1 and 4, Table 2).

**Fig. 5:**
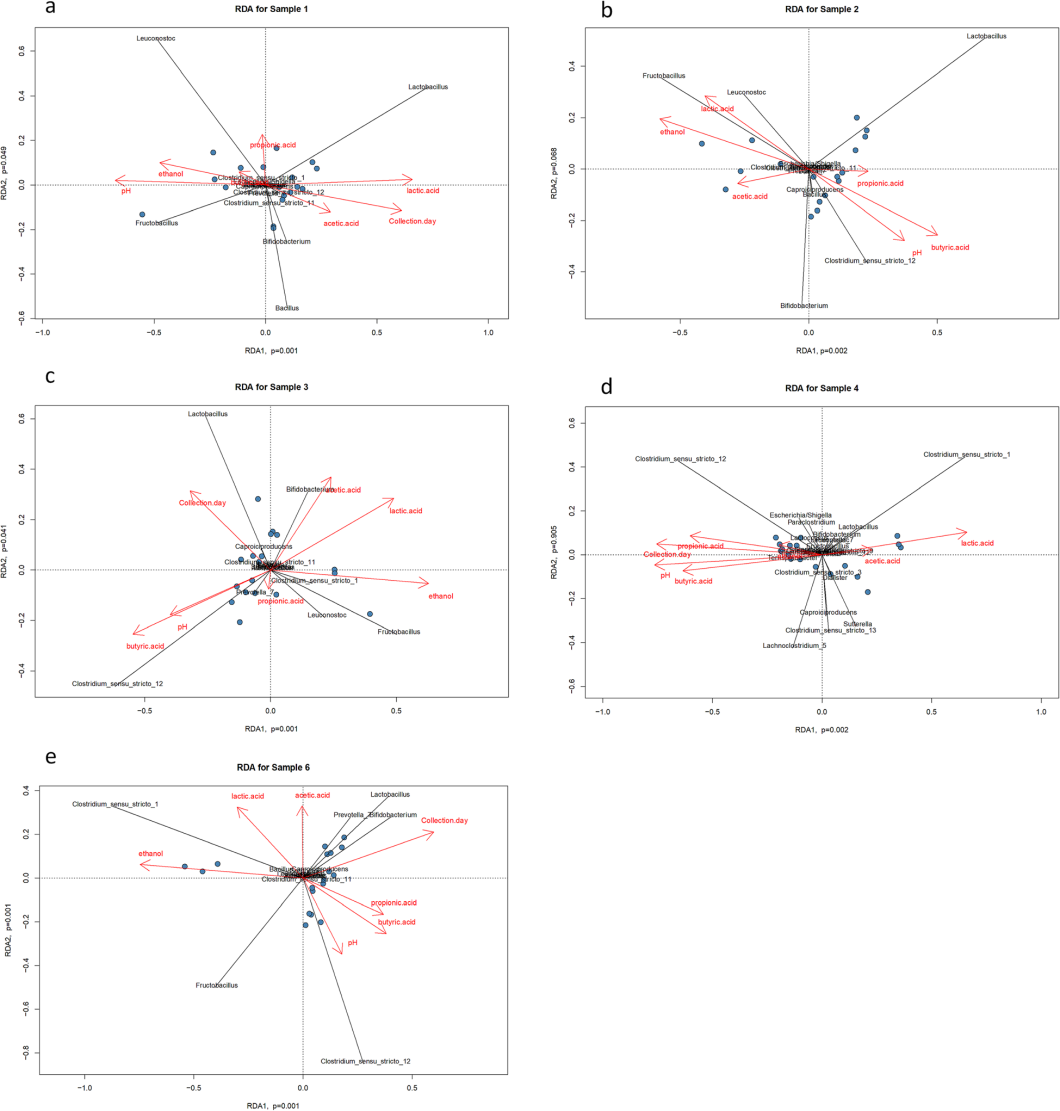
The correlations between the non-gaseous fermentation products, pH of the digestive liquids and the dominant bacterial taxa presented as redundancy analysis (RDA) for the batch experiments: a. Experiment M, b. Experiment ML, c. Experiment MLA, d. Experiment LA, e. Experiment SLA

To integrate the targeted metabolomic data with the analyses of sample biodiversity, we performed redundancy analysis (RDA), a direct gradient analysis technique which summarises linear relationships between components of response variables that are “redundant” with (i.e. “explained” by) a set of explanatory variables. The results of RDA analysis and the correlation between the fermentation products, the pH of the digestive liquids and the dominant bacterial genera in the respective experiments are presented in Fig. 5. The following positive correlations were observed: C*lostridium* sensu_stricto_12 with butyrate and pH (Experiments ML, MLA, SLA); *Fructobacillus* and *Leuconostoc* with lactate and ethanol (Experiments ML); *Fructobacillus*, *Leuconostoc* and *Clostridium* sensu_stricto_1 with ethanol (Experiment MLA); *Clostridium* sensu_stricto_1 with lactate and ethanol (Experiment SLA) or with lactate and acetate (Experiment LA); *Bifidobacterium* with acetate (Experiments M and ML) or acetate and lactate (Experiment MLA); *Fructobacillus* with ethanol and pH; *Lactobacillus* and lactate (Experiment M); collection day with butyrate and pH (Experiment LA). It is noteworthy that *Lactobacillus* correlated with lactate only in Experiment M.

As a synthesis of our observations, two main scenarios for MCs fermenting sucrose-containing media (Experiments M, ML, MLA, SLA) can be proposed: (i) The low pH of the digestive liquids (< 4) is associated with lactate and ethanol as the main non-gaseous fermentation products. Under such conditions, the production of butyrate is very low. MCs are dominated with LAB (especially *Lactobacillus*) and lactate- and acetate-producer *Bifidobacterium.* The contribution of *Clostridium* is very low. This scenario is best illustrated by Experiment M and to some extent by Experiment ML (till the 12th day). (ii) In the second scenario, illustrated by Experiments MLA and SLA, butyrate dominates among the non-gaseous fermentation products and the pH of the fermentation process is in the range 5–6. Lactate and ethanol are the minor products. The *Clostridium* genus constitutes at least 25% of the MC.

Samples collected late (on days 15 and 18) in Experiment ML indicate an intermediate state between both scenarios. In these conditions, lactate is still the dominant fermentation product and the concentration of ethanol decreases, while butyrate production increases and the pH of the digestive liquids reach 4.5–4.6. This corresponds with a higher contribution of *Clostridium* in the MCs. In all scenarios, propionate remains a minor product during the experiments, a decreasing contribution of *Fructobacillus* is observed over time, and *Lactobacillus* remains to be an abundant genus. Butyrate formation is related to pH increase, higher contribution of *Clostridia* (e.g. *Clostridium* sensu stricto 12) in the MC and an increase in biodiversity that is especially prominent in Experiment LA.

### Carbon balance in the selected static batch experiments

We have previously described an approximate balance of carbon during the fermentation of lactate and acetate to butyrate by *Clostridium butyricum* and proposed a model of lactate/acetate conversion to butyrate [18]. To illustrate metabolic transformations in the batch experiments performed in this study, the approximate millimolar balance of carbon for the selected data from Experiments LA and SLA (as shown in Fig. 4) was calculated and presented in Table 3. The selection criterion was butyrate concentration in the digestive liquids, low on day 3 and high on day 6 in both experiments. The carbon balances are based on the concentrations of sucrose (Experiment SLA only), acetate, lactate, propionate, butyrate and ethanol in the media and the digestive liquids. The calculations take into account (i) concentrations of the remaining non-fermented sucrose in the digestive liquids (~3 millimoles of carbon) which were subtracted from the initial amount of sucrose in the media; (ii) the concentrations of the yeast extract-derived butyrate (18 mmol of carbon) and propionate (3 mmol of carbon) in the media that were subtracted from the butyrate and propionate detected in the fermentation products.

**Table 3:** The approximate balance of carbon for the selected data from experiments LA and SLA based on the concentration of the media components and fermentation products (mean from three replicates shown)

	Experiment	Medium		Fermentation products
Balance 1	Experiment LA after the first passage (3 days)	200 lactate + 85 acetate	→	112 lactate + 49 butyrate + 69 acetate + 3 propionate + 4 ethanol + X (27 millimoles of carbon)**
Balance 2	Experiment LA after the second passage (6 days)	200 lactate + 85 acetate	→	8 lactate + 140 butyrate + 56 acetate + 4 propionate + 4 ethanol + X (73 millimoles of carbon)**
Balance 3	Experiment SLA after the first passage (3 days)	200 lactate + 85 acetate + 347 sucrose	→	345 lactate + 22 butyrate + 113 acetate + 250 ethanol*
Balance 4	Experiment SLA after the second passage (6 days)	200 lactate + 85 acetate + 343 sucrose	→	22 lactate + 318 butyrate + 8 acetate + 14 propionate + 46 ethanol + X (220 millimoles of carbon)**

* The excess of millimoles of carbon in the fermentation products likely comes from the inoculum

** X are estimated bacterial biomass and other products including carbon dioxide

### Shotgun metagenomic analysis of the selected MCs

**Fig. 6:**
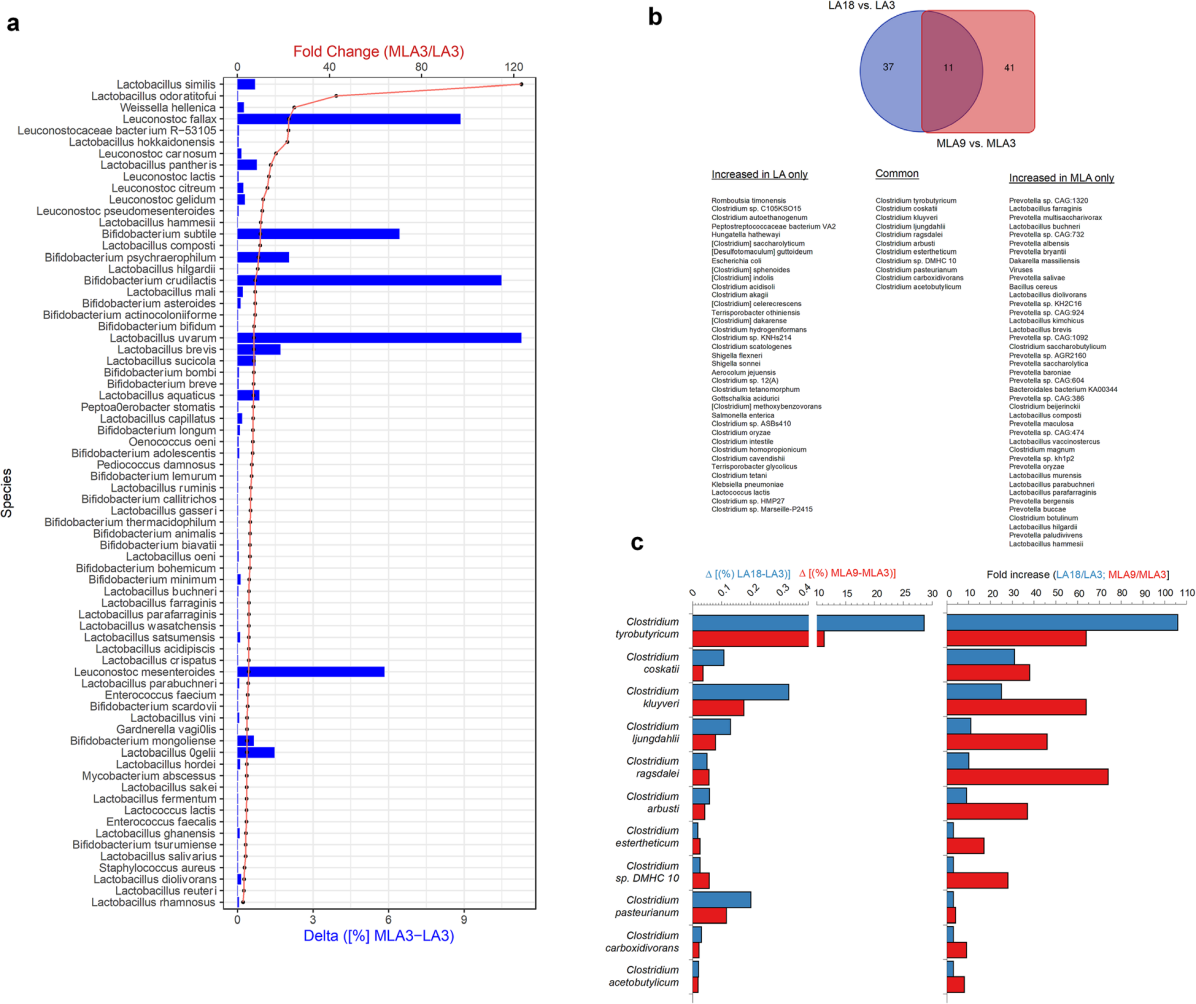
Putatively relevant species producing lactate and butyrate based on metagenomic analysis of the selected MCs: **a.** main lactate producers from molasses fermentation based on species increased over time in sample MLA3 vs LA3 (> 2-fold higher in MLA3, > 0.02% abundance in MLA3); **b.** a Venn diagram main butyrate producers based on species increased over time in sample MLA9 vs MLA3 (in a red square, > 2-fold higher in MLA9, > 0.02% abundance in MLA9) and in sample LA18 vs LA3 (in a blue circle, > 2-fold higher in LA18, > 0.02% abundance in LA18); **c.** The percentage increase (Δ) between two timepoints and the fold change for commonly increased taxa for MLA and LA samples (taxa from the intersection of the Venn diagram from panel **b**). For detailed calculations see Additional file 7

For a better understanding of the dynamics of the MCs and explanation of the observed differences in their metabolic activity, we selected samples from the static batch experiments designated as MLA-3-AC, MLA-9-AC, LA-3-BC, LA-18-AB (summarized in Table 4) and subjected them to shotgun metagenomics analysis. MLA-3-AC is derived from the MLA Experiment (pooled replicates A and C) after the first passage (day 3); when the main non-gaseous fermentation products were lactate and ethanol, the concentration of butyrate was very low (Fig. 4, Additional file 4). Sample MLA-9AC is also derived from the MLA Experiment (pooled replicates A and C) but after the third passage (day 9), when lactate was efficiently utilized and the main fermentation product was butyrate (Fig. 4, Additional file 4). Sample LA-3-BC comes from the LA Experiment (pooled replicates B and C) after the first passage (day 3) when lactate was partially metabolized (Fig. 4, Additional file 4). Sample LA-18-AB comes from the LA Experiment (pooled replicates A and B) after the sixth passage (day 18) when lactate was efficiently utilized and the main fermentation product was butyrate (Fig. 4, Additional file 4). A total of 34,545,964 to 78,144,622 reads per sample was obtained. Taxonomic composition of the MCs on the level of phylum, class, family and genus are presented in Additional file 5. For detailed taxonomic assignments, see Additional file 6. Metagenomic analysis confirmed the results obtained by 16S rRNA sequencing. The goal of this analysis was to identify species potentially responsible for sucrose, acetate and lactate utilization. However, due to limitations of the approach we chose, we limited the data interpretation to two aspects. Since the MLA community produces initially (on day 3) a large quantity of lactate, the first goal was to identify the putative main lactate producers from sucrose (molasses) fermentation. The species more highly represented in MLA3 vs LA3 communities (> 2-fold higher in MLA3, > 0.02% abundance in MLA3) were selected and 72 species that may be involved in the fermentation of sucrose to lactate were identified (Fig. 6, Additional file 7). They were the *Lactobacillus*, *Leuconostoc*, *Bifidobacterium*, *Weissella*, *Enterococcus*, *Gardnerella*, *Pediococcus*, *Oenococcus* and *Peptoaerobacter* species. The top species were *Lactobacillus uvarum* (7-fold higher in MLA3, ∆ = 11.3%), *L. brevis* (7-fold higher in MLA3, ∆ = 1.7%), *Leuconostoc fallax* (22-fold higher in MLA3, ∆ = 8.9%), *L. mesenteroides* (5-fold higher in MLA3, ∆ = 5.8%), *Bifidobacterium crudilactis* (8-fold higher in MLA3, ∆ = 10.5%) and *B. subtile* (10-fold higher in MLA3, ∆ = 6.4%).

**Table 4:** Summary of the selected samples used for shotgun metagenomic sequencing

Sample ID	Experiment	Day of passage/replicates	Sample name in Fig. 3	Fermentation substrate /products description
MLA-3-AC	MLA	3/pooled A+C	MLA 3 A and MLA 3 C	The main non-gaseous fermentation products of molasses were lactate and ethanol, concentration of butyrate was very low
MLA-9-AC	MLA	9/pooled A+C	MLA 9 A and MLA 9 C	The main fermentation product of molasses supplemented with acetate and lactate was butyrate; lactate as a substrate was efficiently utilized
LA-3-BC	LA	3/pooled B+C	LA 3 B and LA 3 C	Lactate as a substrate was partially metabolized
LA-18-AB	LA	18/pooled A+B	LA 18 A and LA 18 B	The main fermentation product of the medium containing exclusively lactate and acetate was butyrate; lactate as a substrate was efficiently utilized

The second aspect of the analysis was a comparison of LA3 vs. LA18 and MLA3 vs. MLA9 MCs to find lactate and acetate utilizers and butyrate producers (Fig. 6, Additional file 7). The species more highly represented in MLA9 vs MLA3 communities (> 2-fold higher in MLA9, > 0.02% abundance in MLA9) were selected and 52 species were identified (Fig. 6, Additional file 7). They were mostly the *Clostridium* and *Prevotella*, as well as *Lactobacillus*, *Dakarella* and *Bacillus* species. The top species was *C. tyrobutyricum* (64.5-fold higher in MLA9, ∆ = 11.3%). Interestingly, in comparison to MLA-3-AC in the sample MLA-9-AC, a decreased contribution of *Leuconostoc* (below 1%) was observed whereas *Lactobacillus* (*L. uvarum* 12.4%, *L. brevis* 4.4%) and *Bifidobacterium* (*B. crudilactis* 3.3% and *B. subtile* 12.9%) were still the top species.

The species more highly represented in LA18 vs LA3 communities (> 2-fold higher in LA18, > 0.02% abundance in LA18) were selected and 48 species were identified (Fig. 6, Additional file 7). They were mostly the *Clostridium*, *Terrisporobacter* as well as *Romboutsia*, *Shigella*, *Aerocolum*, *Gottschalkia*, *Klebsiella* and *Lactococcus* species. The top species were *Clostridium tyrobutyricum* (106-fold higher in LA18, ∆ = 28.6%) and *Terrisporobacter glycolicus* (7.2-fold higher in LA18, ∆ = 4.1%) suggesting that these species contributed to butyrate synthesis. Interestingly, In the LA-3-BC sample (low butyrate formation) the top species were *Clostridium sulfidigenes* (7%) and *Clostridium beijerinckii* (4%). The former was maintained at the level 4.8% whereas the latter dropped to 0.3% in the LA-18-AB sample.

Finally, the common species between MLA9 and LA18 communities were found. All of them were the *Clostridium* species (the most abundant *C. tyrobutyricum* and minor *C. coskatii*, *C. kluyveri*, *C. ljungdahlii*, *C. ragsdalei*, *C. arbusti*, *C. estertheticum*, *Clostridium* sp. DMHC 10, *C. pasteurianum*, *C. carboxidivorans* and *C. acetobutylicum*) (Fig. 6, Additional file 7). There are putatively the most involved in butyrate formation independent on the growth medium (MLA or LA).

**Fig. 7:**
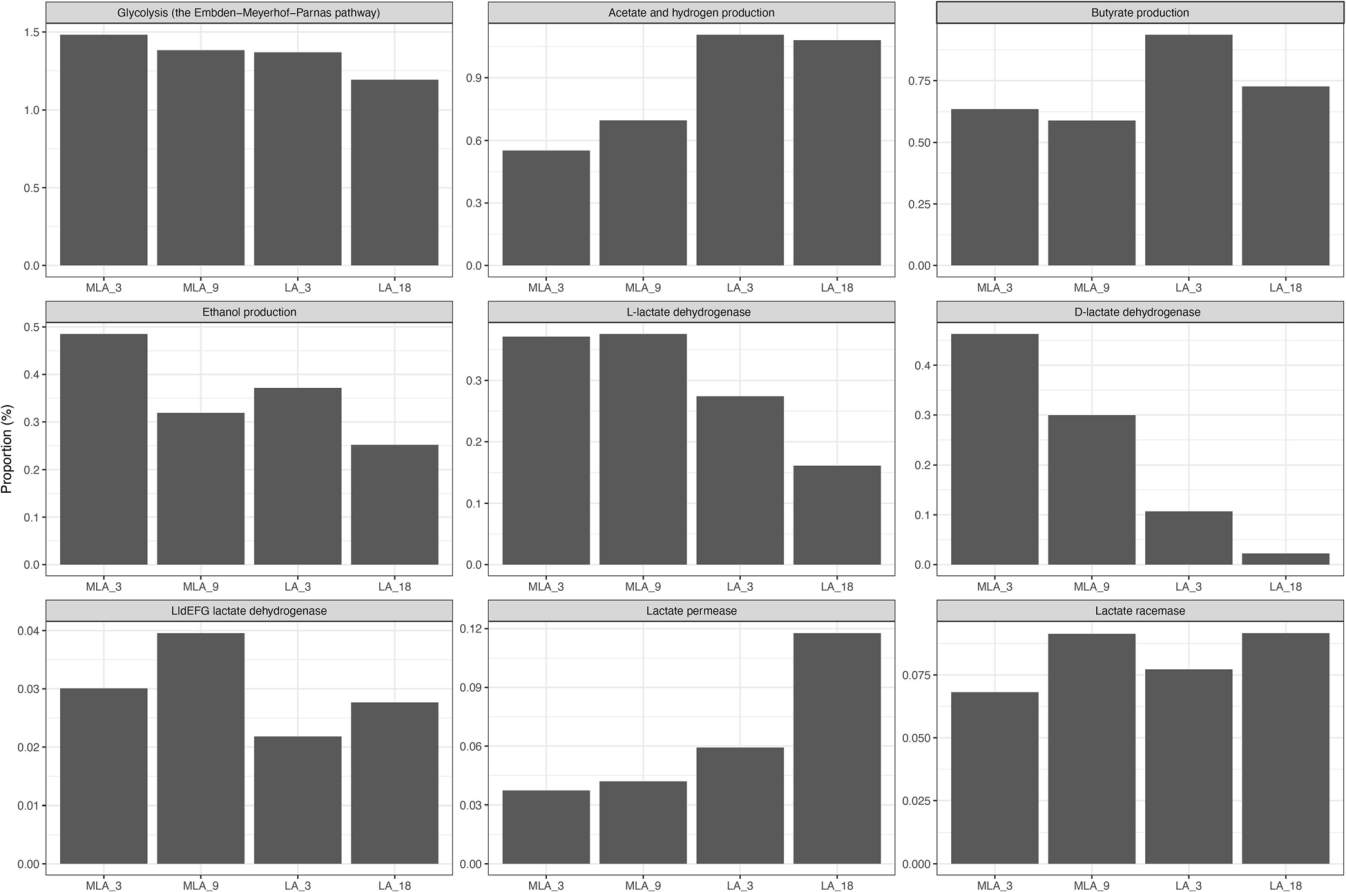
Metabolic potential based on KEGG analysis of the MLA3, MLA9, LA3 and LA18 MCs—the relative abundance of predicted functional genes related to acid fermentation routes. Glycolysis: glucose-6-phosphate isomerase (EC:5.3.1.9, K01810); 6-phosphofructokinase (EC:2.7.1.11, K00850); fructose-bisphosphate aldolase (EC:4.1.2.13, K01623); triosephosphate isomerase (EC:5.3.1.1, K01803); glyceraldehyde 3-phosphate dehydrogenase (EC:1.2.1.12, K00134); phosphoglycerate kinase (EC:2.7.2.3, K00927); enolase (EC:4.2.1.11, K01689); pyruvate kinase (EC:2.7.1.40, K00873). Acetate and hydrogen production: pyruvate-ferredoxin/flavodoxin oxidoreductase (EC:1.2.7.1, EC:1.2.7.-), K03737; ferredoxin hydrogenase gamma subunit (EC:1.12.7.2), K06441; phosphate acetyltransferase (EC:2.3.1.8), K00625; acetate kinase (EC:2.7.2.1), K00925. Butyrate production: acetyl-CoA C-acetyltransferase (EC:2.3.1.9), K00626; 3-hydroxybutyryl-CoA dehydrogenase (EC:1.1.1.157), K00074; phosphate butyryltransferase (EC:2.3.1.19), K00634; butyrate kinase (EC:2.7.2.7), K00929. Ethanol production: acetaldehyde dehydrogenase/alcohol dehydrogenase (EC:1.2.1.10 and EC:1.1.1.1), K04072. Lactate metabolism:l-lactate dehydrogenase (EC:1.1.1.27), K00016; d-lactate dehydrogenase (EC:1.1.1.28), K03778; LldEFG lactate dehydrogenase:l-lactate dehydrogenase complex protein LldG, K00782;l-lactate dehydrogenase complex protein LldE, K18928;l-lactate dehydrogenase complex protein LldF, K18929; Lactate permeases: lactate permease, K03303;l-lactate permease, K00427; Lactate racemase (EC:5.1.2.1), K22373

The functional analysis (KEGG) of the whole communities was conducted to identify selected genes/pathways putatively involved in the fermentation routes related to the MLA and LA conditions (Fig. 7, Additional file 8). A comparable glycolytic potential (Glycolysis, the Embden-Meyerhof-Parnas pathway) was observed for all the examined communities. Overrepresentation of the genes encoding acetaldehyde dehydrogenase/alcohol dehydrogenase in the MLA9 community coincided with the highest concentration of ethanol in the digestive liquids. The lowest contribution of the ethanol synthesis pathway was observed for the LA18 community. The metabolic pathways leading to acetate and hydrogen formation were overrepresented in the MC grown in medium containing exclusively acetate and lactate (samples LA3 and LA18) in comparison to the samples MLA3 and MLA9 grown with the additional sucrose from molasses. Genes encoding enzymes of butyrate synthesis pathways were not overrepresented in the communities producing the highest amount of butyrate (MLA9 ad LA18) suggesting the comparable capacity for butyrate production of all the examined communities. There was a tendency towards an increased contribution of genes encoding lactate permeases responsible for lactate transport [50, 51] in the MCs utilizing lactate, especially when the media contained exclusively lactate and acetate (LA18). Analysis of the abundance of *larA* gene encoding lactate racemase revealed that both MCs grown in MLA and LA media had a similar potential to interconvert the d- and l-enantiomers of lactic acid [52]. In both cases, a slight increase of the potential was observed for the conditions of efficient lactate utilization and butyrate production (MLA3 vs. MLA9 and LA3 vs. LA18). l-lactate dehydrogenase (EC:1.1.1.27) and d-lactate dehydrogenase (EC:1.1.1.28) are responsible for lactate production/consumption. Relative abundance of the genes encoding both these enzymes was lower in the metagenomes of MCs grown in media containing exclusively acetate and lactate (LA3 and LA18) in comparison to those that fermented sucrose (MLA3 and MLA9). Contributions of the *ldhA* gene encoding d-lactate dehydrogenase (EC:1.1.1.28) and the *ldh* gene encodingl-lactate dehydrogenase (EC:1.1.1.27) were higher in the MLA metagenomes compared to the LA metagenomes. In the LA samples, the relative abundance of both genes decreased with the ability to lactate utilization and butyrate synthesis (LA3 vs. LA18) whereas in the MLA samples a decreasing tendency was observed only for the *ldhA* gene encoding d-lactate dehydrogenase. Functional analysis of MLA and LA samples confirmed the presence of the genes encoding LldEFG lactate dehydrogenase recognized as lactate oxidizing enzyme [50]. Their contribution showed an increasing tendency with the capabilities of the MCs to lactate utilization and butyrate production (MLA3 vs MLA9 and LA3 vs LA18). The methodological tool used did not allow for detection of genes encoding the enzymatic complex lactate dehydrogenase (NAD^+^,ferredoxin) (EC1.3.1.110) indentified in *Acetobacterium woodii* [53].

## Discussion

This study describes the dynamics of the metabolic activity and the structure of MCs sampled from hydrogen-producing DF bioreactor and tested in static batch experiments. The results contribute to a better understanding of the dynamics and plasticity of DF MCs that are key factors responsible for the stability and instability of the hydrogen production process. This study continues and expands our previous research [18] on lactate and acetate conversion to butyrate by the bacteria of DF. Here we provide new data on (i) the conditions that favour and unfavour transformation of lactate and acetate to butyrate by DF MCs and (ii) the key players of the MCs determining ability to the conversion process. The study involved five independent static batch experiments performed under anaerobic conditions differing from the media subjected to fermentation. The media contained molasses as a source of sucrose or pure sucrose with the addition of lactate and acetate, or exclusively a mixture of lactate and acetate. Molasses has been used in our studies for hydrogen and methane production in a two-stage process [49, 54, 55]. Sucrose is an attractive substrate for glycolytic fermentations. Lactate and acetate are substrates for butyrate, hydrogen and carbon dioxide formation. Previously, we have shown that DF MCs are unable to grow on lactate only-containing media [18].

The experiments in this study focused on the analysis of non-gaseous fermentation products and the examination of the MCs by 16S rDNA profiling complemented by metagenomics analysis of the selected samples from Experiments MLA and LA (Additional files 5 and 6). Changes in the MCs and selection of the specific groups of bacteria during the batch experiments point to the significance of substrates and metabolic activity of bacteria for the community structure and plasticity. It is noteworthy that the differences were also observed between replicates of the same experiments. This variability revealed in the batch experiments may explain the unstable operation of DF bioreactors observed in many studies [9]. It may also illustrate metabolic microniches that can be formed in the bioreactors.

In the Experiments M, ML, MLA and SLA when the media contained a source of sucrose, stimulation of bacterial growth, especially of lactic acid bacteria, was observed compared to the Experiment LA where the only substrates were lactate and acetate. Chatellard and co-workers [56] found that when the mixed bacterial communities were grown in the presence of different types of carbohydrates, hexoses promoted LAB whereas pentoses stimulated the growth of hydrogen producers. Sucrose is a disaccharide composed of glucose (hexose) and fructose (pentose) which theoretically provides equal conditions for the growth of both groups of bacteria. However, other studies revealed that LAB display both a higher growth rate and a higher ability of substrate uptake than Clostridia which allows them to out-compete other fermentative bacteria [57, 58].

The first 3 days of all batch experiments regardless of the substrate were characterized by a high concentration of lactate and a low concentration of butyrate. MCs processing molasses-containing media (Experiments M, ML, MLA) consisted mainly of lactate, ethanol and acetate producers. The metagenomic analysis of the MLA MC on day 3 revealed that the top species were *Lactobacillus uvarum*, *L. brevis*, *Bifidobacterium subtile*, *B. crudilactis*, *Leuconostoc fallax* and *L.mesenteroides.* This period (day 3) resembles the first stage of hydrogen production from tequila vinasse and nixtamalization wastewater in batch co-fermentation experiments done by Garcia-Depraect et al. [20]. In their studies, the majority of fermentable carbohydrates were metabolized to lactate by *Lactobacillus*, *Sporolactobacillus* and *Streptococcus* and acetate by *Acetobacter* that dominated in the MCs. Lactate and acetate were further used for the production of butyrate and hydrogen by hydrogen-producing bacteria [19, 20]. In our Experiments SLA and LA (the latter with no carbohydrates in the medium), after the first 3 days, the most abundant genus was *Clostridium* sensu stricto 1, unlikely to convert lactate to butyrate.

Our observations concerning MCs selected in Experiment M confirm the commonly recognized fact about the replacement of clostridial-type fermentation by lactic acid fermentation in hydrogen-producing bioreactors and support the thesis about the negative role of lactic acid bacteria in the DF MCs [9, 33, 59]. Analysis of the non-gaseous fermentation products in digestive liquids from Experiment M revealed that besides lactic acid, the dominant product was also ethanol, both products of heterolactic fermentation. Butyric acid was a minor product and a drop in pH to < 4.0 was observed. Noteworthy was a high concentration of ethanol that together with a low pH (< 4.0) might be a relevant factor responsible for the metabolic shift of MC towards lactic acid fermentation and maintenance of its stable metabolic activity. We recently published similar observations of disturbances in hydrogen-producing bioreactors [33, 60]. Previously, we have also shown butyrate formation by the DF MCs grown on the medium containing only molasses [18]. However, compared to this study, the previous media contained a higher concentration of Na_2_HPO_4_ and KH_2_PO_4_, which increased their buffering capacity and helped to maintain pH at 5.

The presence of external lactate or lactate and acetate in the medium was a stimulating factor for the growth of butyrate producers (Experiments ML, MLA and SLA). Lactate and acetate are also fermentation products. Our results clearly show that the balance between lactic acid bacteria and butyrate producers is key for the conversion of lactate and acetate to butyrate. DF MCs that the most effectively converted lactate and acetate to butyrate (Experiments SLA and MLA) were composed of *Clostridium* sensu stricto 12, *Lactobacillus*, *Fructobacillus*, *Bifidobacterium* and *Prevotella.* A summary of the batch experiments where lactate and acetate were transformed to butyrate clearly shows a significant consumption of lactate (or its low concentration) among the non-gaseous products despite the presence of lactic acid bacteria in the MCs. It is in accordance with the previous studies where the effluents from hydrogen-producing bioreactors and the MCs were examined [49, 54, 61]. It can also explain a lack or a weak correlation between *Lactobacillus* and lactate in Experiments ML, MLA and SLA. Interestingly, Esquivel-Elizondo and co-workers [62] considered *Lactobacillaceae* a putative butyrate producer. Other authors have also proposed such a hypothesis (for review see [58]).

It is noteworthy that *Clostridium* sensu stricto 12 was an abundant taxon in all butyrate-producing MCs. Metagenomic analysis of the selected samples from Experiments MLA (day 9) and LA (day 18) revealed a significant contribution of *Clostridium tyrobutyricum* in the MCs. *C. tyrobutyricum* is a recognized hydrogen- and butyrate-producing bacterium via conversion of lactate and acetate [31, 32].

Previously, we have postulated that pH may be a critical factor responsible for a balance of DF MCs. In our experiments, pH was established and maintained intrinsically in the flasks with no additional pH adjustments. Lactate and acetate were transformed to butyrate at pH ≈ 7 when the substrate did not contain carbohydrates and or 5–6 when the substrate contained molasses or pure sucrose. Other studies reported pH in the range of 5.5–6.5 as optimal for hydrogen production and butyrate formation from lactate and acetate [13, 21, 24, 30–32, 61].

Interestingly, Garcia-Depraect et al. [21] reported that an increase of pH above 6.5 caused domination of *Blautia* and *Propionicum* genera in the MC processing tequila vinase and nixtamalization wastewater, and a metabolic shift leading to propionate production. However, in our study, low concentrations of propionate were detected within the non-gaseous fermentation products in all the samples. Propionate-type fermentation characteristic of, e.g. *Clostridium propionicum* [34], thus seemed irrelevant.

On one hand, the results of our research confirm that lactic acid bacteria compete with DF bacteria and inhibit their growth [9–13]. However, the putative role of ethanol as the promoting factor is novel. Ethanol and a low pH may be thought to provide unfavourable conditions for butyrate producers and conversion of lactate and acetate to butyrate. On the other hand, our results strongly support the thesis that conversion of lactate and acetate to butyrate is a common process in DF bioreactors. Furthermore, it is believed that this metabolic pathway is the main route of hydrogen production during acidic fermentation of carbohydrate-rich substrates [19–25]. In our previous paper, we have reported that a community balance between hydrogen producers and lactic acid bacteria is the key factor for stable hydrogen-producing systems. The metabolic shift to lactic acid fermentation or solventogenic pathways reduced hydrogen production [33]. Further investigations should concentrate on the search for communication mechanisms (including quorum-sensing) regulating functioning of DF MCs, also with regard to pH and ethanol contribution. The regulation seems to be more complex than maintaining lactate and acetate transformation and likely includes mutual metabolic stimulation of bacteria. One of the barriers to industrial-scale application of the DF method of hydrogen production is insufficient knowledge of the mechanisms of cooperation between specific groups of bacteria in DF MCs. This issue has been discussed in the recent excellent review by Garcia-Depraect and co-workers [58], on biohydrogen production from lactate.

Although during the batch test in this study the hydrogen production was not measured, we calculated the balances of carbon for selected time points. The balances of carbon performed for Experiment SLA differ dependently on the contribution of butyrate formation. The X value (meaning bacterial biomass and other fermentation products including fermentation gases) was higher when the transformation of lactate and acetate to butyrate was observed. Since the bacterial biomass was similar in every experiment, the differences included fermentation gases and eventually other non-analysed products. The balance of carbon for the transformation of lactate and acetate to butyrate in Experiment LA was similar to that for the pure culture of *Clostridium butyricum* [18] with regard to millimoles of butyrate. The formation of butyrate was not limited by propionate synthesis. A lower concentration of ethanol within the non-gaseous fermentation products in comparison to the previous study [18] may have resulted from a lower concentration of acetate in the medium and/or activity of other bacteria within the community. It should be noted that acetate is a substrate and an intermediate on the pathway of lactate to butyrate transformation [15, 28, 29].

It is noteworthy that the biodiversity of MCs measured by the taxonomic richness and evenness increased with the capability to transform lactate into butyrate. This capacity was lowest when the MCs were dominated by lactic acid bacteria and the most abundant fermentation products were lactate and ethanol. Additionally, the highest biodiversity was observed in MCs grown in the presence of lactate and acetate (Experiment LA) and was related to the presence of taxa not found in other Experiments. These include *Terrisporobacter*, *Lachnoclostridium*, *Paraclostridium* or *Sutterella.* The *Paraclostridium* strain CR4 was isolated from sugar cane bagasse involved in hydrogen and butyric acid production [63]. Other genera were found in and were isolated from human intestine microbiome [64–67].

Since lactic acid and butyric acid fermentations are ubiquitous and some analogies can be found between anaerobic digesters and the mammalian gut, our results are also relevant in the context of human microbiome analyses. Interestingly, studies on the microbiomes from the patients with autism spectrum disorder revealed an increased number of *Terrisporobacter*, *Lachnoclostridium* [66]. *Lachnoclostridium* was identified as a novel bacterial marker for the non-invasive diagnosis of colorectal adenoma [67]. It was also found that most of the gut butyrate-producing bacteria were significantly decreased in patients with non-small-cell lung cancer compared to healthy adults [68]. Also, diarrhoea is often associated with the accumulation of lactate in the hindgut in the case of intestinal disorders such as short-bowel syndrome, inflammatory bowel disease, ulcerative colitis, dyspepsia antibiotic-associated diarrhoea [69].

## Conclusions

The batch tests revealed dynamics of metabolic activity and relevant differences in the composition of DF MCs dependent on fermentation conditions. These results expand our knowledge on lactate to butyrate conversion by DF MCs and are relevant for understanding the processes inside hydrogen-producing bioreactors. The MCs unable to butyrate formation are dominated by lactic acid bacteria. The main fermentation products are lactate and ethanol; the drop of pH to < 4.0 is observed. Further investigations should concentrate on the role of pH and ethanol in the changes of the MC structure and metabolic shifts towards lactate fermentation.

With the ability to convert lactate and acetate to butyrate, the biodiversity of MCs increases. The process of conversion proceeds at pH ≈ 5–6 when the media contain carbohydrates. The most relevant for lactate to butyrate conversion is the balance between lactic acid bacteria (mainly *Lactobacillus*) and butyrate producers (especially *Clostridium* sensu stricto 12, also *Prevotella)*. Mutual metabolic stimulation of bacteria is likely and should not be discounted. In the absence of carbohydrates, the process of converting lactate and acetate to butyrate proceeds at pH ~ 7 and the most abundant bacteria belong to the clostridial species. The increased contribution of *Terrisporobacter*, *Lachnoclostridium*, *Paraclostridium* or *Sutterella* is also observed*.* Metagenomic analysis revealed *C. tyrobutyricum* as the most abundant *Clostridium* species in the butyrate-producing MCs fermenting media with and without carbohydrates.

## Supplementary Information

**Additional file 1.** Statistical analysis of bacterial growth (OD_600nm_), pH of the digestive liquids and the non-gaseous fermentation products. The STATISTICA (version 10.0) computer software (StatSoft, Inc.) was used. All variables were examined for normality and homogeneity of variance. Tukey's HSD (honestly significant difference) test was applied after ANOVA analysis to compare statistical significance among the variables in experiments. Statistical significance was considered at p < 0.05.

**Additional file 2.** Detailed taxonomic composition (genus level) of the MCs selected in batch experiment based on hypervariable V4 region of the 16S rRNA gene, sequenced on MiSeq platform (Illumina).

**Additional file 3.** Alpha Diversity (a. Shannon, b. Simpson) of the MCs selected in time in the static batch experiments for each collection day, except day 0, which is an inoculation day. The lower and upper hinges represent the first and third quartiles respectively. The whiskers extends to the largest and lowest values. The middle line represents the median value.

**Additional file 4.** Detailed characteristics of the digestive liquids from the batch experiments – data used for preparation of Figures 3, 4 and Table 2. The samples from which the material was taken for shotgun metagenomic sequencing are marked in yellow.

**Additional file 5.** Taxonomic composition of the MCs from the selected samples (see Table 4) based on shotgun metagenomic sequencing analysis: a. phylum level, b. class level, c. family level, d. genus level. Only the top 10 most dominant lineages are shown, the remaining lineages and the unclassified sequences are grouped into other. For detailed taxonomic assignments see Additional File 6.

**Additional file 6.** Detailed taxonomic composition of the MCs from the selected samples (see Table 4) based on shotgun metagenomic sequencing analysis: phylum, class, order, family, genus, species.

**Additional file 7.** Detailed calculations for Figure 6 based on taxonomic assignments in Additional File 6.

**Additional file 8.** Detailed data describing metabolic potential based on KEGG analysis of the MLA3, MLA9, LA3 and LA18 MCs - the relative abundance of predicted functional genes related to acid fermentation routes.

## Data Availability

All data generated or analysed during this study are included in this published article [and its supplementary information files]. The raw DNA sequences generated in this study have been deposited in NCBI databases with the accession numbers PRJNA645198 (16SrRNA) and PRJNA640235 (shotgun metagenomics sequencing).

## Abbreviations

AD: Anaerobic digestion
DF: Dark fermentation
Experiment M: Experiment with molasses
Experiment ML: Experiment with molasses plus sodium lactate
Experiment MLA: Experiment with molasses plus sodium lactate and sodium acetate
Experiment SLA: Experiment with sucrose plus sodium lactate and sodium acetate
Experiment LA: Experiment with sodium lactate and sodium acetate
HPLC: High-performance liquid chromatography
LAB: Lactic acid bacteria
MCs: Microbial communities
NMDS: Non-metric multidimensional scaling analysis
OD: Optical density
SCFAs: Short-chain fatty acids
SD: Standard deviation
Tukey’s HSD: Tukey’s honestly significant difference test

## References

[1] Thauer RK, Kaster AK, Seedorf H, Buckel W, Hedderich R (2008). Methanogenic archaea: ecologically relevant differences in energy conservation. Nat Rev Microbiol.

[2] Liu Y, Whitman WB (2008). Metabolic, phylogenetic, and ecological diversity of the methanogenic archaea. Ann N Y Acad Sci.

[3] Sikora A, Detman A, Chojnacka A, CBaszczyk M. Anaerobic digestion: I. A common process ensuring energy flow and the circulation of matter in ecosystems. II. A tool for the production of gaseous biofuels. In: Angela Faustino Jozala, editor. Fermentation Processes. Rijeka, Croatia: InTech; 2017. p. 271-301.

[4] Angenent LT, Karim K, Al-Dahhan MH, Wrenn BA, Domíguez-Espinosa R (2004). Production of bioenergy and biochemicals from industrial and agricultural wastewater. Trends Biotechnol.

[5] Hallenbeck PC (2005). Fundamentals of the fermentative production of hydrogen. Water Sci Technol.

[6] Das D, Veziroglu TN (2008). Advances in biological hydrogen production processes. Int J Hydrog Energy.

[7] Seth EC, Taga ME (2014). Nutrient cross-feeding in the microbial world. Front Microbiol.

[8] Daeschel M, Andersson RE, Fleming H (1987). Microbial ecology of fermenting plant materials. FEMS Microbiol Rev.

[9] Etchebehere C, Castelló E, Wenzel J, del Pilar A-RM, Borzacconi L, Buitrón G (2016). Microbial communities from 20 different hydrogen-producing reactors studied by 454 pyrosequencing. Appl Microbiol Biotechnol.

[10] Noike T, Takabatake H, Mizuno O, Ohba M (2002). Inhibition of hydrogen fermentation of organic wastes by lactic acid bacteria. Int J Hydrog Energy.

[11] Ren N, Xing D, Rittmann BE, Zhao L, Xie T, Zhao X (2007). Microbial community structure of ethanol type fermentation in bio-hydrogen production. Environ Microbiol.

[12] Sreela-Or C, Imai T, Plangklang P, Reungsang A (2011). Optimization of key factors affecting hydrogen production from food waste by anaerobic mixed cultures. Int J Hydrog Energy.

[13] Palomo-Briones R, Trably E, López-Lozano NE, Celis LB, Méndez-Acosta HO, Bernet N, Razo-Flores E (2018). Hydrogen metabolic patterns driven by Clostridium-Streptococcus community shifts in a continuous stirred tank reactor. Appl Microbiol Biotechnol.

[14] Moens F, Verce M, De Vuyst L (2017). Lactate-and acetate-based cross-feeding interactions between selected strains of lactobacilli, bifidobacteria and colon bacteria in the presence of inulin-type fructans. Int J Food Microbiol.

[15] Duncan SH, Louis P, Flint HJ (2004). Lactate-utilizing bacteria, isolated from human feces, that produce butyrate as a major fermentation product. Appl Environ Microbiol.

[16] Bourriaud C, Robins R, Martin L, Kozlowski F, Tenailleau E, Cherbut C (2005). Lactate is mainly fermented to butyrate by human intestinal microfloras but inter-individual variation is evident. J Appl Microbiol.

[17] Muñoz-Tamayo R, Laroche B, Walter E, Doré J, Duncan SH, Flint HJ, Leclerc M (2011). Kinetic modelling of lactate utilization and butyrate production by key human colonic bacterial species. FEMS Microbiol Ecol.

[18] Detman A, Mielecki D, Chojnacka A, Salamon A, Błaszczyk MK, Sikora A (2019). Cell factories converting lactate and acetate to butyrate: Clostridium butyricum and microbial communities from dark fermentation bioreactors. Microb Cell Factories.

[19] García-Depraect O, León-Becerril E (2018). Fermentative biohydrogen production from tequila vinasse via the lactate-acetate pathway: Operational performance, kinetic analysis and microbial ecology. Fuel..

[20] García-Depraect O, Valdez-Vázquez I, Rene ER, Gómez-Romero J, López-López A, León-Becerril E (2019). Lactate-and acetate-based biohydrogen production through dark co-fermentation of tequila vinasse and nixtamalization wastewater: Metabolic and microbial community dynamics. Bioresour Technol.

[21] García-Depraect O, Rene ER, Gómez-Romero J, López-López A, León-Becerril E (2019). Enhanced biohydrogen production from the dark co-fermentation of tequila vinasse and nixtamalization wastewater: novel insights into ecological regulation by pH. Fuel..

[22] García-Depraect O, Rene ER, Diaz-Cruces VF, León-Becerril E (2019). Effect of process parameters on enhanced biohydrogen production from tequila vinasse via the lactate-acetate pathway. Bioresour Technol.

[23] García-Depraect O, Gómez-Romero J, León-Becerril E, López-López A (2017). A novel biohydrogen production process: Co-digestion of vinasse and Nejayote as complex raw substrates using a robust inoculum. Int J Hydrog Energy.

[24] Fuess LT, Zaiat M, do Nascimento CAO (2019). Novel insights on the versatility of biohydrogen production from sugarcane vinasse via thermophilic dark fermentation: Impacts of pH-driven operating strategies on acidogenesis metabolite profiles. Bioresour Technol.

[25] Fuess LT, Júnior ADNF, Machado CB, Zaiat M (2018). Temporal dynamics and metabolic correlation between lactate-producing and hydrogen-producing bacteria in sugarcane vinasse dark fermentation: the key role of lactate. Bioresour Technol.

[26] Schwalm ND, Mojadedi W, Gerlach ES, Benyamin M, Perisin MA, Akingbade KL (2019). Developing a Microbial Consortium for Enhanced Metabolite Production from Simulated Food Waste. Fermentation..

[27] Park J-H, Kim D-H, Baik J-H, Park J-H, Yoon J-J, Lee C-Y, Kim SH (2021). Improvement in H2 production from Clostridium butyricum by co-culture with Sporolactobacillus vineae. Fuel..

[28] Diez-Gonzalez F, Russell JB, Hunter JB (1995). The role of an NAD-independent lactate dehydrogenase and acetate in the utilization of lactate byClostridium acetobutylicum strain P262. Arch Microbiol.

[29] Shen G-J, Annous B, Lovitt R, Jain M, Zeikus J (1996). Biochemical route and control of butyrate synthesis in Butyribacterium methylotrophicum. Appl Microbiol Biotechnol.

[30] Matsumoto M, Nishimura Y (2007). Hydrogen production by fermentation using acetic acid and lactic acid. J Biosci Bioeng.

[31] Wu C-W, Whang L-M, Cheng H-H, Chan K-C (2012). Fermentative biohydrogen production from lactate and acetate. Bioresour Technol.

[32] Jo JH, Lee DS, Park D, Park JM (2008). Biological hydrogen production by immobilized cells of Clostridium tyrobutyricum JM1 isolated from a food waste treatment process. Bioresour Technol.

[33] Detman A, Laubitz D, Chojnacka A, Wiktorowska-Sowa E, Piotrowski J, Salamon A, et al. Dynamics and Complexity of Dark Fermentation Microbial Communities Producing Hydrogen From Sugar Beet Molasses in Continuously Operating Packed Bed Reactors. Front Microbiol. 2021;11(3303). 10.3389/fmicb.2020.612344 PMC7819888 33488554

[34] Baghchehsaraee B, Nakhla G, Karamanev D, Margaritis A (2009). Effect of extrinsic lactic acid on fermentative hydrogen production. Int J Hydrog Energy.

[35] Kim T-H, Lee Y, Chang K-H, Hwang S-J (2012). Effects of initial lactic acid concentration, HRTs, and OLRs on bio-hydrogen production from lactate-type fermentation. Bioresour Technol.

[36] Caporaso JG, Lauber CL, Walters WA, Berg-Lyons D, Huntley J, Fierer N, Owens SM, Betley J, Fraser L, Bauer M, Gormley N, Gilbert JA, Smith G, Knight R (2012). Ultra-high-throughput microbial community analysis on the Illumina HiSeq and MiSeq platforms. ISME J.

[37] Callahan BJ, McMurdie PJ, Rosen MJ, Han AW, Johnson AJA, Holmes SP (2016). DADA2: High-resolution sample inference from Illumina amplicon data. Nat Methods.

[38] Wang Q, Garrity GM, Tiedje JM, Cole JR (2007). Naive Bayesian classifier for rapid assignment of rRNA sequences into the new bacterial taxonomy. Appl Environ Microbiol.

[39] Quast C, Pruesse E, Yilmaz P, Gerken J, Schweer T, Yarza P, Peplies J, Glöckner FO (2013). The SILVA ribosomal RNA gene database project: improved data processing and web-based tools. Nucleic Acids Res.

[40] Oksanen J, Blanchet FG, Friendly M, Kindt R, Legendre P, McGlinn D, Minchin PR, OʼHara RB, Simpson GL, Solymos P, Stevens MHH, Szoecs E, Wagner H (2019). Vegan: Community Ecology Package.

[41] Wickham H (2016). ggplot2: Elegant Graphics for Data Analysis.

[42] Ploner A (2020). Heatplus: Heatmaps with row and/or column covariates and colored clusters. R package version 2.34.0.

[43] Martin M (2011). Cutadapt removes adapter sequences from high-throughput sequencing reads. EMBnet J.

[44] Bolger AM, Lohse M, Usadel B (2014). Trimmomatic: a flexible trimmer for Illumina sequence data. Bioinformatics..

[45] Li D, Liu CM, Luo R, Sadakane K, Lam TW (2015). MEGAHIT: an ultra-fast single-node solution for large and complex metagenomics assembly via succinct de Bruijn graph. Bioinformatics..

[46] Hyatt D, Chen GL, Locascio PF, Land ML, Larimer FW, Hauser LJ (2010). Prodigal: prokaryotic gene recognition and translation initiation site identification. BMC Bioinform.

[47] Li H, Durbin R (2010). Fast and accurate long-read alignment with Burrows-Wheeler transform. Bioinformatics..

[48] Kanehisa M, Sato Y, Kawashima M, Furumichi M, Tanabe M (2016). KEGG as a reference resource for gene and protein annotation. Nucleic Acids Res.

[49] Chojnacka A, Błaszczyk MK, Szczęsny P, Nowak K, Sumińska M, Tomczyk-Żak K, Zielenkiewicz U, Sikora A (2011). Comparative analysis of hydrogen-producing bacterial biofilms and granular sludge formed in continuous cultures of fermentative bacteria. Bioresour Technol.

[50] Pinchuk GE, Rodionov DA, Yang C, Li X, Osterman AL, Dervyn E, Geydebrekht OV, Reed SB, Romine MF, Collart FR, Scott JH, Fredrickson JK, Beliaev AS (2009). Genomic reconstruction of Shewanella oneidensis MR-1 metabolism reveals a previously uncharacterized machinery for lactate utilization. Proc Natl Acad Sci U S A.

[51] Dong JM, Taylor JS, Latour DJ, Iuchi S, Lin EC (1993). Three overlapping lct genes involved in L-lactate utilization by Escherichia coli. J Bacteriol.

[52] Desguin B, Goffin P, Viaene E, Kleerebezem M, Martin-Diaconescu V, Maroney MJ, Declercq JP, Soumillion P, Hols P (2014). Lactate racemase is a nickel-dependent enzyme activated by a widespread maturation system. Nat Commun.

[53] Weghoff MC, Bertsch J, Müller V (2015). A novel mode of lactate metabolism in strictly anaerobic bacteria. Environ Microbiol.

[54] Detman A, Chojnacka A, Błaszczyk M, Kaźmierczak W, Piotrowski J, Sikora A (2017). Biohydrogen and biomethane (Biogas) production in the consecutive stages of anaerobic digestion of molasses. Pol J Environ Stud.

[55] Chojnacka A, Szczęsny P, Błaszczyk MK, Zielenkiewicz U, Detman A, Salamon A, Sikora A (2015). Noteworthy facts about a methane-producing microbial community processing acidic effluent from sugar beet molasses fermentation. PLoS ONE.

[56] Chatellard L, Trably E, Carrère H (2016). The type of carbohydrates specifically selects microbial community structures and fermentation patterns. Bioresour Technol.

[57] Rombouts JL, Kranendonk EMM, Regueira A, Weissbrodt DG, Kleerebezem R, van Loosdrecht MCM (2020). Selecting for lactic acid producing and utilising bacteria in anaerobic enrichment cultures. Biotechnol Bioeng.

[58] García-Depraect O, Castro-Muñoz R, Muñoz R, Rene ER, León-Becerril E, Valdez-Vazquez I, Kumar G, Reyes-Alvarado LC, Martínez-Mendoza LJ, Carrillo-Reyes J, Buitrón G (2021). A review on the factors influencing biohydrogen production from lactate: the key to unlocking enhanced dark fermentative processes. Bioresour Technol.

[59] Sikora A, Błaszczyk M, Jurkowski M, Zielenkiewicz U, Kongo JM (2013). Lactic acid bacteria in hydrogen-producing consortia: on purpose or by coincidence?. Lactic Acid Bacteria - R & D for Food, Health and Livestock Purposes.

[60] Detman A, Chojnacka A, Mielecki D, Błaszczyk MK, Sikora A (2018). Inhibition of hydrogen-yielding dark fermentation by ascomycetous yeasts. Int J Hydrog Energy.

[61] Park MJ, Jo JH, Park D, Lee DS, Park JM (2010). Comprehensive study on a two-stage anaerobic digestion process for the sequential production of hydrogen and methane from cost-effective molasses. Int J Hydrog Energy.

[62] Esquivel-Elizondo S, Ilhan Z, Garcia-Peña E, Krajmalnik-Brown R. Insights into butyrate production in a controlled fermentation system via gene predictions. MSystems. 2017;2(4). 10.1128/mSystems.00051-17 PMC5516221 28761933

[63] Rabelo CABS, Okino CH, Sakamoto IK, Varesche MBA (2020). Isolation of Paraclostridium CR4 from sugarcane bagasse and its evaluation in the bioconversion of lignocellulosic feedstock into hydrogen by monitoring cellulase gene expression. Sci Total Environ.

[64] Ohashi Y, Fujisawa T (2019). Analysis of Clostridium cluster XI bacteria in human feces. Biosci Microbiota Food Health.

[65] Sakamoto M, Ikeyama N, Kunihiro T, Iino T, Yuki M, Ohkuma M (2018). Mesosutterella multiformis gen. nov., sp. nov., a member of the family Sutterellaceae and Sutterella megalosphaeroides sp. nov., isolated from human faeces. Int J Syst Evol Microbiol.

[66] Marietta E, Horwath I, Taneja V (2018). Microbiome, immunomodulation, and the neuronal system. Neurotherapeutics..

[67] Liang JQ, Li T, Nakatsu G, Chen Y-X, Yau TO, Chu E (2020). A novel faecal Lachnoclostridium marker for the non-invasive diagnosis of colorectal adenoma and cancer. Gut..

[68] Gui Q, Li H, Wang A, Zhao X, Tan Z, Chen L, et al. The association between gut butyrate-producing bacteria and non-small-cell lung cancer. J Clin Lab Anal. 2020:e23318. 10.1002/jcla.23318 PMC7439349 32227387

[69] Hashizume K, Tsukahara T, Yamada K, Koyama H, Ushida K (2003). Megasphaera elsdenii JCM1772T normalizes hyperlactate production in the large intestine of fructooligosaccharide-fed rats by stimulating butyrate production. J Nutr.

