# Supplementary material for: Dynamics of dark fermentation microbial communities in the light of lactate and butyrate production
Source: Microbiome. 2021 Jul 14;9:158. doi: 10.1186/s40168-021-01105-x (PMC8281708; doi:10.1186/s40168-021-01105-x)
Supplement: Supplementary file 4 — Additional file 3. Alpha Diversity (a. Shannon, b. Simpson) of the MCs selected in time in the static batch experiments for each collection day, except day 0, which is an inoculation day. The lower and upper hinges represent the first and third quartiles respectively. The whiskers extends to the largest and lowest values. The middle line represents the median value. [file 40168_2021_1105_MOESM4_ESM.pptx]

## Slide 1
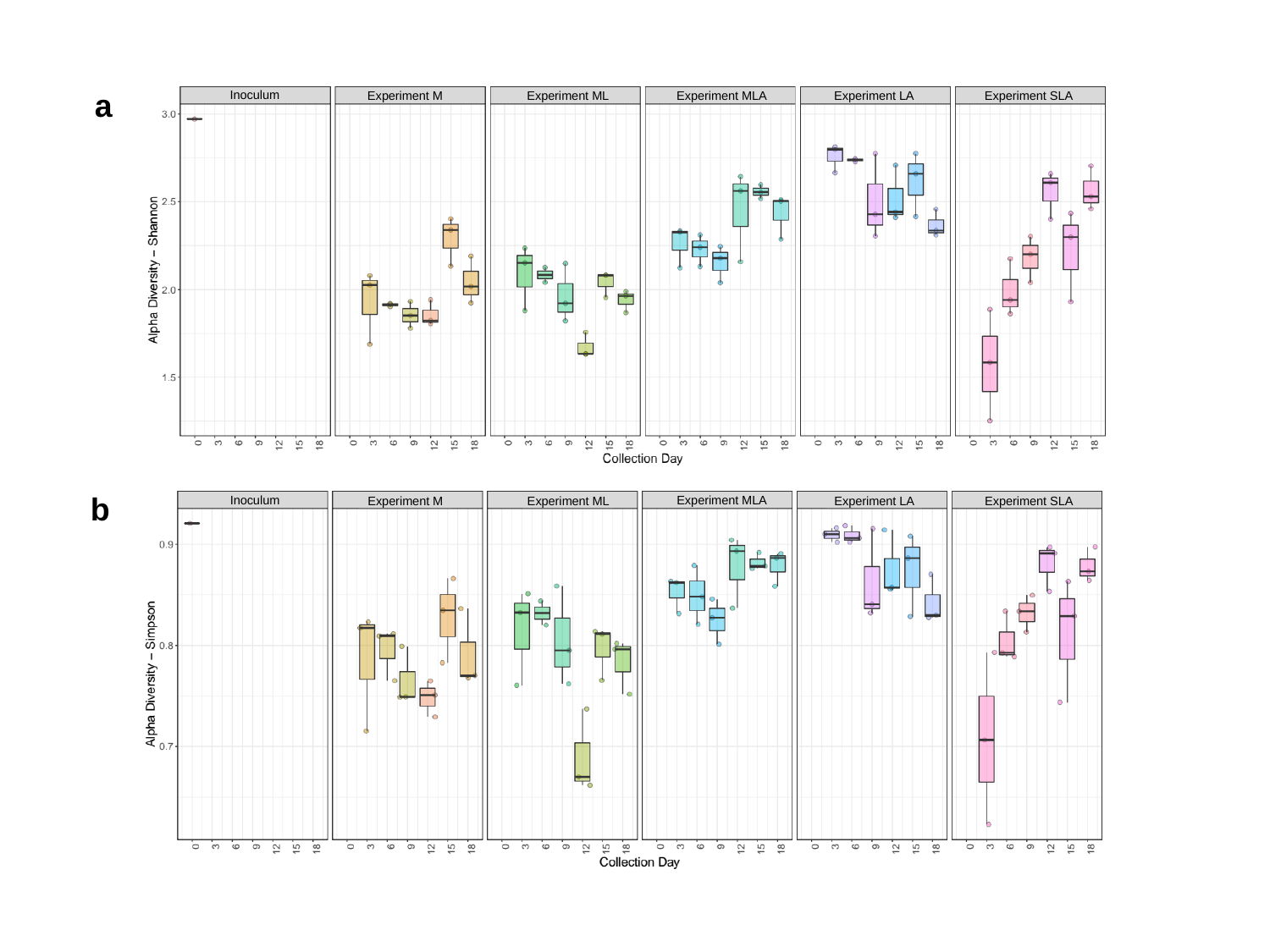

a
Inoculum
Experiment MLA
Experiment SLA
Experiment ML
Experiment LA
Experiment M
b
Inoculum
Experiment MLA
Experiment SLA
Experiment ML
Experiment LA
Experiment M
