# Supplementary figures and images for: Dynamics of dark fermentation microbial communities in the light of lactate and butyrate production
Source: Microbiome. 2021 Jul 14;9:158. doi: 10.1186/s40168-021-01105-x (PMC8281708; doi:10.1186/s40168-021-01105-x)

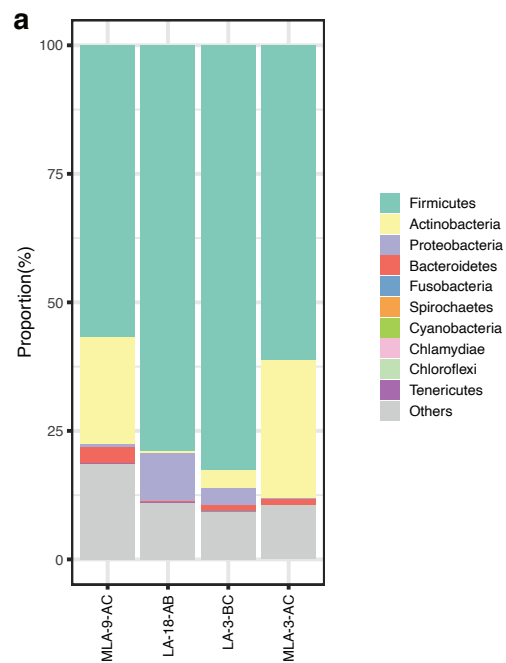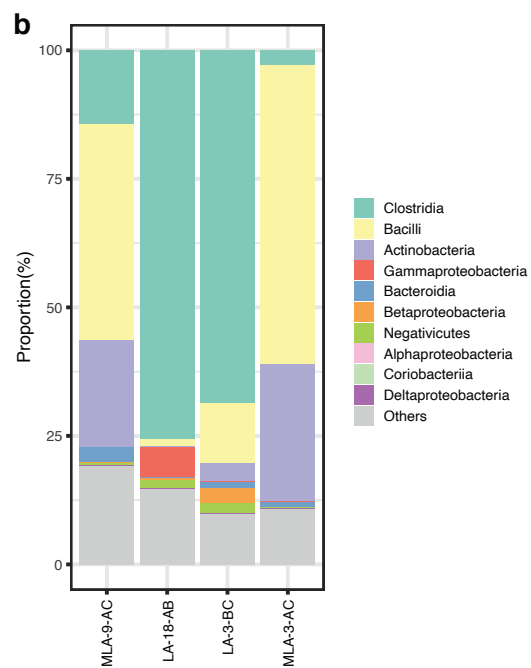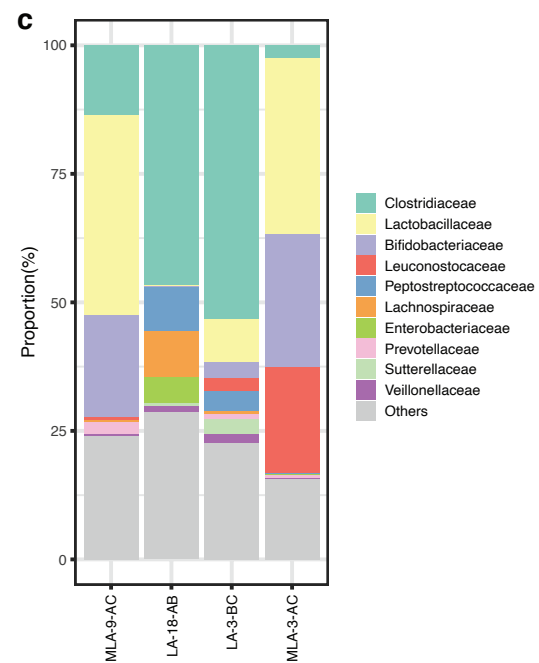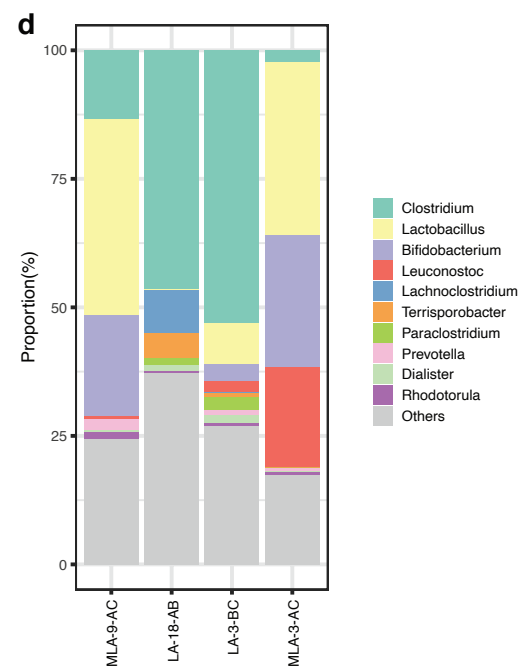

Supplement: Supplementary file 6 — Additional file 5. Taxonomic composition of the MCs from the selected samples (see Table 4) based on shotgun metagenomic sequencing analysis: a. phylum level, b. class level, c. family level, d. genus level. Only the top 10 most dominant lineages are shown, the remaining lineages and the unclassified sequences are grouped into other. For detailed taxonomic assignments see Additional File 6. [file 40168_2021_1105_MOESM6_ESM.pdf]
